# A chemotherapy response prediction model derived from tumor-promoting B and Tregs and proinflammatory macrophages in HGSOC

**DOI:** 10.3389/fonc.2023.1171582

**Published:** 2023-07-14

**Authors:** Yue Xi, Yingchun Zhang, Kun Zheng, Jiawei Zou, Lv Gui, Xin Zou, Liang Chen, Jie Hao, Yiming Zhang

**Affiliations:** ^1^ Department of Reproductive Medicine, Central Hospital Affiliated to Shandong First Medical University, Jinan, Shandong, China; ^2^ Department of Urology, Shanghai Sixth People’s Hospital Affiliated to Shanghai Jiao Tong University School of Medicine, Shanghai, China; ^3^ Institute of Clinical Science, Zhongshan Hospital, Fudan University, Shanghai, China; ^4^ Department of Pathology, Jinshan Hospital, Fudan University, Shanghai, China; ^5^ Jinshan Hospital Center for Tumor Diagnosis & Therapy, Jinshan Hospital, Fudan University, Shanghai, China; ^6^ Department of Gynecological Oncology, Shandong Cancer Hospital Affiliated to Shandong University, Shandong Academy of Medical Sciences, Jinan, China

**Keywords:** chemotherapy, single-cell RNA-seq, high-grade serous ovarian cancer (HGSOC), bioinformatics, response prediction model

## Abstract

**Background:**

Most patients with high-grade serous ovarian cancer (HGSOC) experienced disease recurrence with cumulative chemoresistance, leading to treatment failure. However, few biomarkers are currently available in clinical practice that can accurately predict chemotherapy response. The tumor immune microenvironment is critical for cancer development, and its transcriptomic profile may be associated with treatment response and differential outcomes. The aim of this study was to develop a new predictive signature for chemotherapy in patients with HGSOC.

**Methods:**

Two HGSOC single-cell RNA sequencing datasets from patients receiving chemotherapy were reinvestigated. The subtypes of endoplasmic reticulum stress-related XBP1^+^ B cells, invasive metastasis-related ACTB^+^ Tregs, and proinflammatory-related macrophage subtypes with good predictive power and associated with chemotherapy response were identified. These results were verified in an independent HGSOC bulk RNA-seq dataset for chemotherapy. Further validation in clinical cohorts used quantitative real-time PCR (qRT-PCR).

**Results:**

By combining cluster-specific genes for the aforementioned cell subtypes, we constructed a chemotherapy response prediction model containing 43 signature genes that achieved an area under the receiver operator curve (AUC) of 0.97 (*p* = 2.1e-07) for the GSE156699 cohort (88 samples). A huge improvement was achieved compared to existing prediction models with a maximum AUC of 0.74. In addition, its predictive capability was validated in multiple independent bulk RNA-seq datasets. The qRT-PCR results demonstrate that the expression of the six genes has the highest diagnostic value, consistent with the trend observed in the analysis of public data.

**Conclusions:**

The developed chemotherapy response prediction model can be used as a valuable clinical decision tool to guide chemotherapy in HGSOC patients.

## 1 Introduction

Ovarian cancer (OC) is a carcinoma of the female reproductive system that has a high incidence and mortality rate (1). Ovarian cancer is insidious in its early stages, with no distinct symptoms and an unknown location, and approximately 70% of patients are already in the late stage when diagnosed (2). High-grade serous ovarian cancer (HGSOC) is the most frequent phenotype among all ovarian cancer subtypes, accounting for approximately 60%–80% of cases (3). Most HGSOCs are diagnosed at advanced stages with poor prognoses, and the standard treatment scheme is surgery plus platinum-based chemotherapy (4). However, many patients are initially susceptible to chemotherapy and have clinical relief, but over time, they will develop resistance or relapse. Ovarian cancer can be defined as a platinum-sensitive type [platinum-free interval (PFI) of ≥6 months] or a platinum-resistant type (PFI of <6 months) (5). Ovarian carcinoma patients’ clinical results remain suboptimal due to a lack of biomarkers that can properly predict chemotherapy response/resistance. Reliable predictive models of chemotherapy responsiveness in ovarian cancer can help physicians accurately estimate chemotherapy outcomes and develop regimens that are in the best interest of patients, and personalized treatment will have significant implications for improving patient prognosis (6).

Tumor tissues comprise heterogeneous cell types and tumor microenvironments (TMEs), made up of immune cells, stromal cells, blood vessels/lymphatics, nerve endings, extracellular matrix (ECM), etc. The TME plays a key role in the occurrence, development, and therapeutic response of cancer (7, 8). Abnormal changes in the TME can affect patient prognosis and treatment response (9), and the TME has complicated functions, both in interacting with the immune system to exhibit tumor-killing capacity and in mediating tumor-promoting effects (10). The molecular characteristics of relevant immune cell populations in the TME may reveal therapeutically relevant predictors. The advent of scRNA-seq technology offers the possibility of exploring gene expression profiles at cellular resolution in the TME (11). Previous studies identified several factors associated with chemotherapy outcomes, such as high tumor-infiltrating lymphocyte (TIL) abundance (12), high density of CD8^+^T cells and LAMP3^+^ mature dendritic cells (DCs) (13), high CD4^+^CD68^+^CD20^+^ and low CD8^+^ T-lymphatic infiltration (14), and tumor-infiltrating immune cell (TIIC) abundance (15), correlated with chemotherapy response. Tumor-associated macrophages (TAM) (16) or hyperinflation of tumor-associated stromal cells (17), activation of the endoplasmic reticulum stress pathway (18), and defects in cell death pathways (19) can lead to chemotherapy resistance. The study by Pierluigi Giampaolino et al. also summarized the biomarkers associated with the clinical outcome of ovarian cancer, such as CA125 and HE4 (20). However, the exact mechanism of resistance has not been fully determined, and although several studies have been published on the prediction of response to chemotherapy in ovarian cancer, the predictive performance is not high, and effective clinical predictive tools are still lacking (21–24).

In this study, we performed a comprehensive analysis of two independent HGSOC single-cell RNA sequencing (scRNA-seq) datasets (25, 26) to dissect the molecular characteristics of immune cells to construct predictive models. We found that B cells and regulatory T cells could promote chemoresistance, while macrophages were associated with the chemo-response. Based on the above findings, we constructed a chemo-response prediction signature, whose predictive performance was validated in multiple independent bulk RNA-seq datasets. Through further analysis, six genes were found to have the most diagnostic value, EEF1A1, RPL35A, RPL31, RPL11, RPS12, and RPS23, which were further validated by qPCR. Overall, these findings expand our understanding of the factors influencing the chemotherapy response and provide a more accurate response prediction model for the clinical management of ovarian cancer.

## 2 Materials and methods

### 2.1 Dataset collection

Two publicly available HGSOC scRNA-seq datasets were obtained from the Gene Expression Omnibus (GEO) database (https://www.ncbi.nlm.nih.gov/geo/). The GSE165897 dataset (25) was collected from 11 HGSOC patients before and after chemotherapy with prospective tissue samples, and according to the Chemotherapy Response Score (CRS) (27), 3 of the 11 patients were ineffective, and the other 8 were effective after chemotherapy. The GSE154600 dataset (26) contained five independent OC specimens, two of which were chemotherapy-resistant patients, one was a chemorefractory patient, and the remaining two were chemotherapy-sensitive patients. Detailed clinicopathological information for all patients in these two single-cell datasets and the processing, clustering, and cell-type definition methods for the scRNA-seq datasets were described in detail in original articles (25, 26).

The reanalysis results were validated in multiple HGSOC bulk RNA-seq datasets. The bulk RNA-seq datasets used for validation were GSE156699 (22), GSE30161 (28), GSE63885 (29), GSE23554 (30), GSE51373 (31), GSE15622 (32), GSE28739 (33), NACT_Pre (34), and chemo_pre_RPKM (35) from the GEO dataset.

The databases used in this study included MSigDB v7.5.1 (36) (http://www.gsea-msigdb.org/gsea/index.jsp), from which 436 hallmark, KEGG, GOBP, and Reactome gene sets were downloaded for GSEA. The detailed metadata for the scRNA-seq and bulk RNA-seq datasets used in this study and can be found in **Supplementary Table 1**.

### 2.2 Study design

**Figure 1:**
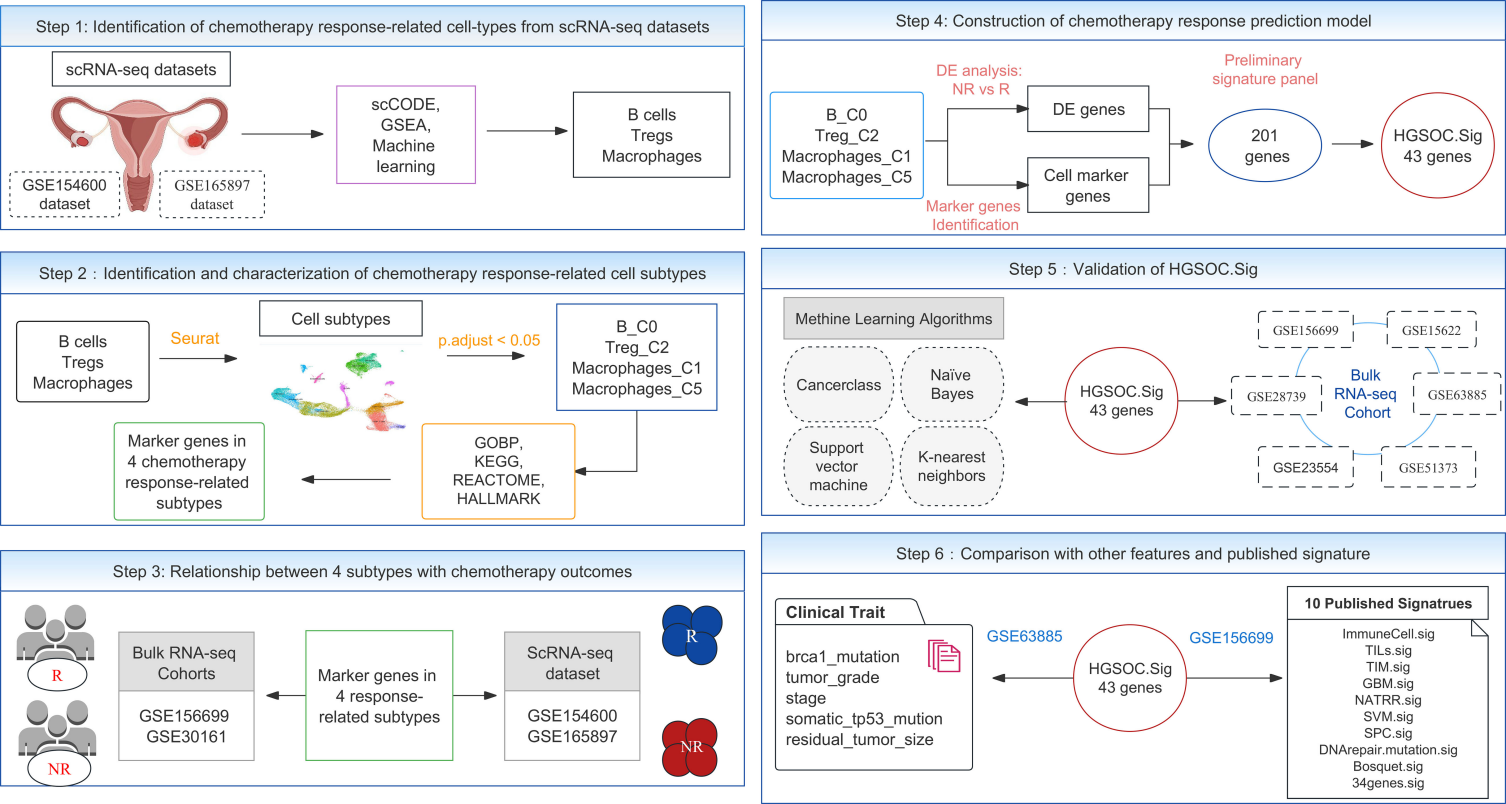
The overall experimental design flowchart. GSE154600 and GSE165897 are scRNA-seq datasets. The bulk RNA-seq datasets included seven cohorts, GSE30161, GSE156699, GSE28739, GSE23554, GSE51373, GSE63885, and GSE15622. GSEA, gene set enrichment analysis; DE, differentially expressed.

The overall design of this study is shown in **Figure 1**. We extracted 10 and 9 cell types from the two scRNA-seq datasets. We then identified differentially expressed (DE) genes in responders (R) and nonresponders (NR) in each of these cells separately using the scCODE (v1.2.0.0) R package (37). scCODE allows the detection of selected DE genes by multiple assays, improving the accuracy of single-cell DE analysis. In this way, for each cell type, two DE gene lists significantly highly expressed in R and NR were obtained, which led to a total of 38 DE gene lists (see Extended Data 1). The investigate gene set tool was used to calculate the enriched gene sets between the obtained gene lists and the gene sets in the Molecular Signature Database (MSigDB). Each DE gene list was sorted by the absolute value of its log fold change (LogFC) value in descending order. Gene sets enriched in GOBP, Hallmark, KEGG, and Reactome were identified by submitting the top 500 genes (if less than 500, all genes were submitted). Using the default settings of MSigDB [showing the top 10 gene sets and false discovery rate (FDR) *p*-values less than 0.05], each gene list was enriched in several gene sets. The predictive power of these gene sets was then examined with the Cancerclass (v1.40.0) R package (38). In order to adhere to the limitations imposed by the Cancerclass (v1.40.0) R package, which requires a minimum of three genes as input, gene sets containing fewer than three genes were excluded from the analysis. This decision was made to ensure the compatibility of the dataset with the package’s requirements and to maintain the validity and integrity of the subsequent analyses. A final 920 gene sets were obtained for subsequent analysis (see Extended Data 2).

We tested the predictive ability of the 920 gene sets on the outcome of chemotherapy response in the GSE156699 cohort (*n* = 88, R = 50, NR = 38) using Cancerclass. The sensitivity and specificity of the predictions were evaluated using the receiver operating characteristic (ROC) curve and the corresponding area under the ROC curve (AUC). Each gene set was tested as an independent classifier, and the *p*-values of the AUC were calculated by the Welch *t*-test built-in Cancerclass, which reflects the validity of the classification results. These 920 AUC *p*-values (see Extended Data 2) were used for subsequent cell subtype selection.

### 2.3 Data processing for single-cell RNA sequencing

We adopted the cell types defined in the original article for the GSE165897 dataset (25) and reannotated the cell types in the GSE154600 dataset (26). For each cell type, we performed fine-grained clustering using the Seurat (v4.1.1) R package (39). Specifically, we first performed cross-sample integration on the extracted datasets. Prior to this, the expression of each gene was normalized by the total expression in the corresponding cell, multiplied by a scale factor of 10,000, and then log2 transformed (Seurat default setting). The 2,000 variable features were identified by indVariableFeature and used for subsequent analysis. The batch effect was then corrected using the mutual nearest neighbor (MNN) method in the Batchelor (v1.12.3) R package (40), and the percentage of mitochondrial transcripts was regressed using ScaleData. The combined analysis was used for dimension reduction and clustering only, and raw log-normalized expression data were used for all DE and gene-level analyses. RunPCA was used to perform principal component analysis on the output of the combined analysis. The first 20 principal components were used to perform Louvain clustering of cells with a resolution parameter of 0.5. Finally, visualization was performed in two dimensions using Uniform Flow Profile Approximation and Projection (UMAP) (Dims = 1:20). This procedure was used for all cell types analyzed for scRNA-seq datasets.

### 2.4 Differential expression gene analysis

DE gene analysis was performed on each Louvain cluster and all other clusters using FindAllMarkers built into the Seurat package by setting the parameters min.pct = 0.1 and logfc.threshold = 0.25. Genes with *p*.adjust < 0.05 were selected as cluster-specific marker genes. The R package scCODE (v1.2.0.0) was used to analyze the DE genes of responders and nonresponders in each cell type. In all DE analyses, genes with *p*-values < 0.05 corrected by Bonferroni FDR were considered DE genes by the bilateral Wilcoxon rank sum test.

### 2.5 GSVA and GSEA

We used the Gene Set Variation Analysis (GSVA) method with default settings to assign a specific gene signature activity score to individual cells or samples, as implemented in the GSVA (v1.38.2) R package (41). GSEA was performed on preordered DE gene lists based on predownloaded Hallmark, KEGG, GOBP, and Reactome gene sets using the default parameters of the ClusterProfiler (v4.4.4) R Package (42). In addition, this R package provides the functionality to investigate whether a specific gene set exhibits enrichment at either the upper or lower portions of a gene list that has been prearranged. The significance of enrichment was determined by evaluating gene sets with FDR-corrected *p*-values below 0.05, utilizing the Benjamini–Hochberg method, in the context of a comparative analysis between two groups. Consequently, gene sets meeting this criterion were regarded as significantly enriched in one group when compared to the other.

### 2.6 Survival analysis, Cox, and logistic regression analysis

Survival analysis using the Kaplan–Meier method and univariate and multivariate Cox regression analyses were performed on GSVA scores for specific gene signatures. The analysis was conducted using the Survival (v3.3.1) R package (43) and SurvMiner (v0.4.9) R package (44). To classify the “high-score” and “low-score” groups within the GSE30161 cohort, we employed a dichotomization method based on the median GSVA score. To compare the groups, an analysis of variance (log-rank) test was employed. Logistic regression analysis was performed using the Survminer R package, utilizing the built-in GLM function, to assess the relationship between chemotherapy outcomes and the average expression of specific gene signatures. To visualize the relevant receiver operating characteristic (ROC) curves, the pROC (v1.18.0) R package was utilized.

### 2.7 Chemotherapy response prediction signature development workflow

Based on the cluster-specific genes of four cell subtypes, XBP1^+^ B cells, ACTB^+^ Tregs, FCN1^+^ macrophages, and CCL3^+^ macrophages, we developed gene signatures using Cancerclass according to the process shown in **Figure 1**. The GOBP genes of these four cell subtypes were condensed using Cancerclass to obtain the *p*-values of the ROC curves, which were then FDR-adjusted using the Benjamini–Hochberg method (see Extended Data 5). We merged all gene sets with *p*.adjust < 0.05 (38 gene sets in total) to obtain a total of 770 genes (union.genelist). Additionally, we compared the changes in the expression fold change of all genes in R and NR in the corresponding cell subtypes by DE analysis. For XBP1^+^ B, we chose R and NR gene avg_log2FC > −0.1; for ACTB^+^ Tregs, we chose R and NR gene avg_log2FC > −0.1; for FCN1^+^ macrophages, we chose R and NR gene avg_log2FC < 0.1; for CCL3^+^ macrophages, we chose R and NR gene avg_log2FC < 0.1. After intersecting these selected genes, we obtained a gene list containing 11,933 genes (intersect.genelist). Subsequently, we took the intersection of union.genelist and intersect.genelist and excluded the missing genes in the GSE156699 cohort. We ended up with a gene panel of 201 genes. Then, we used this gene panel to run the recursive algorithm. First, we exhausted the 201 combinations by selecting 200 genes out of the 201 gene panel. Next, the predictive capability of all these combinations was examined in the GSE156699 cohort, and the AUC was estimated with Cancerclass. Among these 201 combinations, we retained the gene combination with the highest AUC for the next cycle. For each cycle, the gene panel was reduced by one gene until the number of genes in the gene panel reaches 3, and finally, the highest AUC of the gene panels in all cycles was selected (see Extended Data 6, **Figure S13**). The selected gene panel is referred to as HGSOC.Sig in this study.

### 2.8 Construction of the PPI network and hub gene analysis

STRING v11.5 (https://string-db.org) was used for constructing protein–protein interaction (PPI) networks for genes extracted from a preliminary signature panel (201 genes) with significant predictive power, which was based on the cluster-specific markers for four cell subtypes associated with chemotherapy response. *Homo sapiens* was selected as the organism of interest, the minimum required interaction score was set to high confidence (0.7), and the remaining parameters were used as defaults.

### 2.9 Human research and RNA extraction and quantitative real-time polymerase chain reaction

Tumor tissue samples for the validation experiments were collected from HGSOC patients, including three chemosensitive and three chemoresistant patients. To define chemosensitivity, common definitions of platinum resistance were used, including “resistant” patients who relapsed less than 6 months after cessation of chemotherapy or progressed during treatment (PFI < 6 months), and “sensitive” patients who relapsed 6 months or more after chemotherapy (5). The study was reviewed and approved by the Research Ethics Committee of the Cancer Hospital Affiliated to Shandong First Medical University. All patients/participants provided written informed consent to participate in this study.

All tumor tissues were lysed by RNAex Pro Reagent [Accurate Biotechnology (Hunan) Co., Ltd, Changsha, China] and total RNA was extracted according to the manufacturer’s instructions. Next, a QuickDrop Spectrophotometer nucleic acid protein quantifier (Molecular Devices in Holliston, MA 01746 USA) was used to measure the concentration and purity of the RNA solution. Before qRT-PCR, the extracted RNA was reverse transcribed to cDNA using the Evo M-MLV RT Mix Kit with gDNAClean for qPCR Ver. 2 [Accurate Biotechnology (Hunan), Changsha, China]. The qRT-PCR reaction consisted of 2 μl of reverse transcription product, 7.2 μl of RNase-free water, 10 μl of 2X SYBR^®^ Green Pro Taq HS Premix [Accurate Biotechnology (Hunan), Changsha, China], and 0.4 μl each of forward and reverse primers. PCR was performed in a LightCycler ^®^ 480 II real-time fluorescent quantitative PCR system (Roche Diagnostics, 91115 Hague RoaD, Indianapolis, IN, USA). qRT-PCR was performed using the following primer sequences. The forward primer of EEF1A1 was “TTCGGGCAAGTCCACCACTAC.” The reverse primer of EEF1A1 was “CGCTCAGCTTTCAGTTTATCCAAGA.” The forward primer of RPL35A was “GTTTACGCCCGAGATGAAACAGA.” The reverse primer of RPL35A was “GTTTCCATGGGCCCGAGTTA.” The forward primer of RPL31 was “TCGGGCACTCAAAGAGATTCG.” The reverse primer of RPL31 was “CGGTATGGCACATTCCTTATTCCT.” The forward primer of RPL11 was “CCTGGACTTCTATGTGGTGCTG.” The reverse primer of RPL11 was “CCTCTTTGCTGATTCTGTGTTTGG.” The forward primer of RPS12 was “TCTGAAGACTGCCCTCATCCA.” The reverse primer of RPS12 was “GCCTCCACCAACTTGACATACAT.” The forward primer of RPS23 was “CCAATGACGGTTGCTTGAACT.” The reverse primer of RPS23 was “AGCGGACTCCAGGAATATCAC.” The forward primer of β-action was “TGGCACCCAGCACAATGAA.” The reverse primer of β-action was “CTAAGTCATAGTCCGCCTAGAAGCA.” All primers were synthesized by Accurate Biotechnology [Accurate Biotechnology (Hunan) Co., Ltd, Changsha, China]. The β-actin gene was used as an internal control and the relative expression of six chemotherapy response-related genes was determined using the 2^−ΔΔCt^ method (45). The experiment was repeated in triplicate on independent occasions. Statistical differences in six chemotherapy response-related genes between chemoresistant and chemosensitive samples for ovarian cancer were detected by unpaired *t*-test using GraphPad Prism V8 (GraphPad Software, La Jolla, CA, USA) and tested for statistical significance levels and expressed as *p* < 0.05 for *; *p* < 0.01 for **.

### 2.10 Statistical analysis

The predictive power of each gene panel in the study for chemotherapy response was assessed by drawing ROC plots, computing AUCs, and evaluating the sensitivity and specificity of the implementation with Cancerclass, and the ROC curve’s AUC and *p*-value were used to examine prediction capability. The 95% confidence intervals were computed for sensitivity and specificity via Wilson’s method built in Cancerclass, and *p*-values were calculated via Welch’s *t*-test. Unless otherwise mentioned, all *p*-values in this study were adjusted by the Benjamini–Hochberg method, and adjusted *p*-values < 0.05 were deemed statistically significant. Variables were grouped using Wilcoxon’s test. All confidence intervals were reported as binomial 95% confidence intervals (CIs). All statistical analyses for this study were performed with R (v4.2.1) software.

## 3 Results

### 3.1 B cells, Tregs, and macrophages associated with chemotherapy response/resistance

According to the flowchart in **Figure 1**, we analyzed two scRNA-seq datasets from the GEO database on HGSOC, the GSE154600 dataset (26) containing five samples of patients after neoadjuvant chemotherapy, namely, three chemoresistant and two chemosensitive; and the GSE165897 dataset (25) containing 11 samples of patients after neoadjuvant chemotherapy, namely, 3 chemoresistant and 8 chemosensitive patients. The results were then validated with multiple HGSOC bulk RNA-seq datasets. The details of all scRNA-seq and bulk RNA-seq datasets used in this study are described in *Materials and methods*, and metadata for all data samples can be found in **Supplemental Table 1**.

First, we used the GSE154600 dataset (26) (*n* = 5, R = 2, NR = 3) (**Figure S1A**). There are 10 cell types in the dataset, namely, B cells, CD4^+^ T cells (CD4T), CD8^+^ T cells (CD8T), monocytes, macrophages, natural killer cells (NK), fibroblasts, endothelial cells, mesangial cells, and epithelial cancer cells. The visualization results of the 10 cell types in two dimensions are shown by uniform flow approximation and projection (UMAP) (**Figure S1B**).

DE genes between R and NR in seven immune and fibroblast cell types (excluding cancer cells, mesangial cells, and endothelial cells) were identified using the scCODE R package, yielding 14 DE gene lists (see Extended Data 1). We tried to identify the gene lists that could effectively predict the outcome of chemotherapy response. From the MSigDB, 14 DE gene lists were found to be enriched with multiple functional gene sets, including GOBP, Hallmark, KEGG, and Reactome. The predictive performance of the gene sets was estimated based on receiver operating characteristic (ROC) curves obtained using the Cancerclass R package in the GSE63885 cohort (29) (*n* = 64, R = 54, NR = 10, see *Materials and methods* for details). The AUC *p*-values of these gene sets (see Extended Data 2) are presented in **Figure S2**.

**Figure 2:**
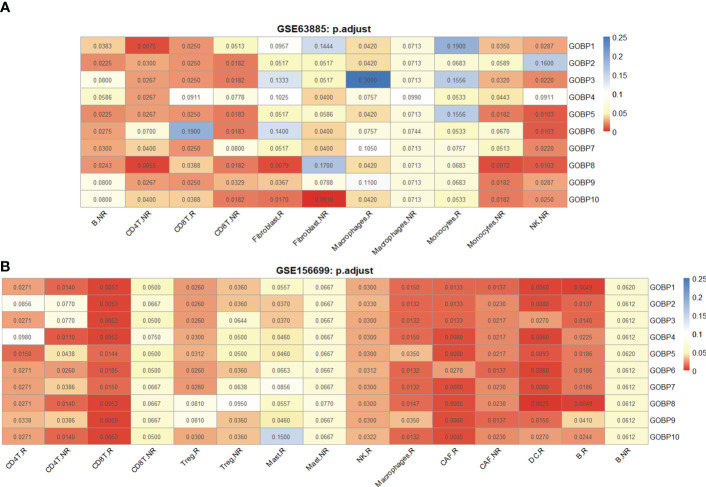
The DE gene lists from different cell types were associated with chemotherapy response prediction. **(A)** Performance of 11 DE gene lists from the GSE154600 dataset in predicting chemotherapy response outcomes. **(B)** Performance of 11 DE gene lists from the GSE154600 dataset in predicting chemotherapy response results. All data for the computational processes can be obtained in detail from Extended Data 1. Chemotherapy response results: R, responders; NR, nonresponders. AUC *p*-values were FDR-adjusted by the Benjamini–Hochberg method.

We found that the GOBP gene sets had overall better predictive performance than Hallmark, KEGG, and Reactome. To examine cell types with good predictive accuracy, we identified the enriched GOBP gene sets with AUC *p*-values < 0.05 (unadjusted) in each DE gene list. A DE gene list was considered significant if no less than half of the top 10 enriched gene sets had AUC *p*-values < 0.05. Next, the significant DE gene lists were identified in Hallmark, KEGG, and Reactome according to the same criteria. As a result, we identified 11 DE gene lists that were relevant for prediction (**Figures S2A–D**). The DE gene lists were further reduced to seven based on FDR-adjusted AUC *p*-values (**Figure 2A**). According to **Figure 2A**, we hypothesized that B cell and macrophage subtypes might be associated with the chemotherapy response.

Similarly, we used the GSE165897 dataset (25) (*n* = 11, R = 8, NR = 3) with cell types defined in the original study (**Figure S1A**). The visualization results of the cell types in two dimensions are shown by UMAP (**Figure S1C**). We analyzed nine cell types in the dataset, namely, B cells Tregs, CD4^+^ T cells, CD8^+^ T cells, macrophages, mast cells, NK cells, cancer-associated fibroblasts (CAFs), and DCs. A total of 18 DE gene lists were obtained using scCODE (**Figures S2E–H**). The functional gene sets enriched by these DE gene lists were identified from MSigDB and evaluated in the GSE156699 cohort (22) (*n* = 88, R = 50, NR = 38) using ROC curves obtained with Cancerclass. The top 10 gene sets that can effectively predict chemotherapy response outcomes were identified based on the criterion of AUC *p*-values < 0.05 for not less than half of the enriched gene sets. As a result, we identified 12 DE gene lists associated with the prediction (FDR-adjusted AUC *p*-values). According to **Figure 2B**, we hypothesized that Tregs and macrophage subtypes may be associated with the chemotherapy response.

### 3.2 XBP1^+^ B and ACTB^+^ Treg cell subtypes are enriched in nonresponders

**Figure 3:**
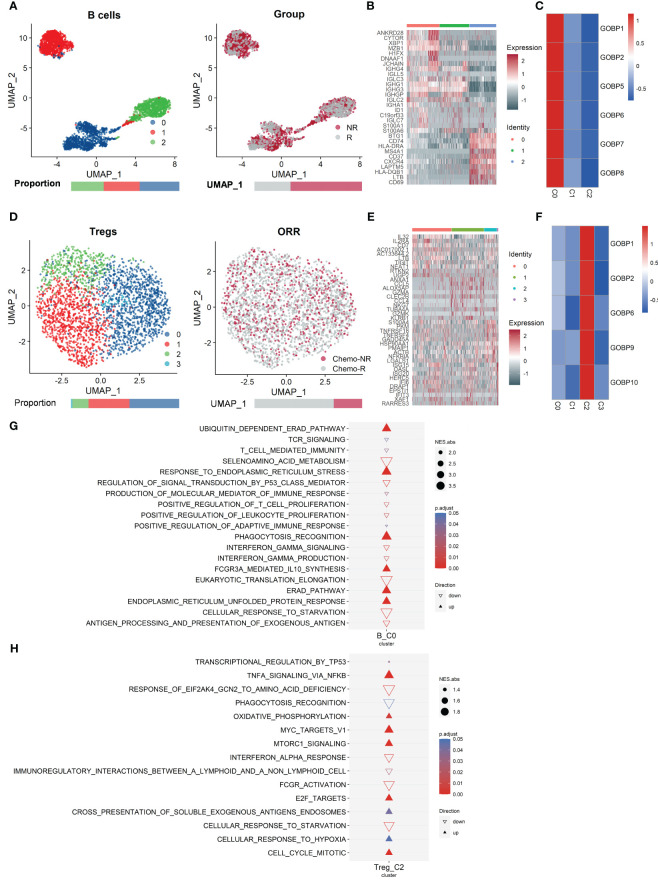
Analysis of the single-cell RNA sequencing dataset GSE154600 versus GSE165897 revealed that B cells from GSE154600 and Tregs from GSE165897 were enriched in nonresponders. **(A)** UMAP plot of B cells from GSE154600. B cells were further divided into three clusters containing two different chemotherapy response outcomes, R and NR. Bar graphs show the proportion of cells sorted by clusters (left) and chemotherapy response (right). **(B)** Heat map of standardized expression of the top 10 specific marker genes of each B-cell subpopulation of GSE154600 as determined by bilateral Wilcoxon rank sum test and FDR correction (*p* < 0.05). **(C)** Expression of GOBP gene sets with significant (*p* < 0.05) predictive power was localized by gene set variance analysis (GSVA) to identify B-cell subtypes associated with chemotherapy response prediction. **(D)** UMAP plot of Tregs from GSE165897. Tregs were further divided into four clusters containing two different chemotherapy response outcomes, Chemo-R and Chemo-NR. The bars show the proportion of cells sorted by clusters (left) and chemotherapy response (right). **(E)** Heat map of standardized expression of the top 10 specific marker genes of each Treg cell subpopulation of GSE165897 as determined by bilateral Wilcoxon rank sum test and FDR correction (*p* < 0.05). **(F)** Expression of GOBP gene sets with significant (*p* < 0.05) predictive power was localized by gene set variance analysis (GSVA) to identify Treg cell subtypes associated with chemotherapy response prediction. **(G, H)** Results of GOBP, Hallmark, KEGG, and Reactome enrichment analyses of the C0 subpopulation of B cells **(G)** from GSE154600 and the C2 subpopulation of Tregs **(H)** from GSE165897.

We further analyzed the list of nonresponder-associated DE genes obtained from the GSE154600 and GSE165897 datasets: B.NR and Treg.NR. The 2,955 B cells (NR.cell = 1963, R.cell = 992) were clustered into three subclusters, and the 2,458 Tregs (NR.cell = 652, R.cell = 1,806) were clustered into four subclusters by Seurat (**Figures 3A, D**). Subsequently, cluster-specific marker genes were identified by FindAllMarkers (Seurat), and the expression heat map of the top 10 marker genes in each cell cluster is shown in **Figures 3B, E**. The classical marker genes of B and Tregs were highly expressed in all subgroups (**Figures S3A, S3B**). Six of GSE154600’s B.NR gene sets and five of GSE165897’s Treg.NR gene sets were found with AUC *p*.adjust < 0.05 (**Figures 2A, B**).

We analyzed the expression of these gene sets by GSVA and found that they were highly expressed in B-cell subcluster 0 (B_C0, NR.cells = 709, R.cells = 375) (**Figures 3C** and **S3C**) and in Treg cell subcluster 2 (Treg_C2, NR.cells = 115, R.cells = 249) (**Figures 3F** and **S3D**). Thus, both B_C0 and Treg_C2 cells may be related to chemoresistance. GOBP, Hallmark, KEGG, and Reactome analyses of these marker genes showed that pathways associated with tumor promotion, such as activation of the ERAD pathway (46, 47), synthesis of the immunosuppressive cytokine IL-10 (48), and drive of endoplasmic reticulum stress (49), were remarkably enriched in B_C0 (**Figures 3G** and **S3G**). In contrast, pathways associated with tumor suppression, such as the P53 signaling pathway (50), antigen delivery and processing (51), and interferon-gamma response (52), were significantly suppressed (**Figure 3G**, see Extended Data 3). In Treg_C2, cell cycle-related pathways (53) and metabolic activities [e.g., oxidative phosphorylation (54)], oncogenic pathways such as MYC (55) and E2F (56) targets were significantly enriched (**Figure 3H**). In contrast, pathways associated with tumor suppression, such as immune activation-related pathways, antigen delivery, recognition and phagocytosis (51), interferon α response (52), antibody binding-related FCGR signaling (57), and complement response excitation pathways, were significantly suppressed in Treg_C2 (**Figure 3H**, see Extended Data 3). Treg_C2 downregulated anticancer interferon α signaling and activated oncogenic MTORC1 signaling (**Figure 3H**). The aforementioned was also supported by their first 20 pathways by normalized enrichment score (NES) (**Figures S3G, H)**.

In addition, we found that in B_C0, genes related to tumor proliferation, migration, and endoplasmic reticulum stress, such as TUBB, TUBA1B (58), CYTOR (59), and XBP1 (60), had higher expression than other clusters (**Figure S3E**). Compared with other clusters, the Treg_C2 subcluster had high expression of genes related to tumor proliferation and metabolism, as well as invasion and metastasis, such as TUBB, TUBA1B, S100A4 (61), HSP90AA1 (62), and ACTB (63) (**Figure S3F**). Because of the high expression of B_C0 for endoplasmic reticulum stress-related markers and Treg_C2 for invasive metastasis-related pathway markers, we annotated B_C0 and Treg_C2 as XBP1^+^ B and ACTB^+^ Treg, respectively.

These findings are consistent with prior research indicating that malfunction of immune-related pathways frequently leads to changes in the tumor immunological milieu and tumor formation. Thus, XBP1^+^ B and ACTB^+^ Tregs lead to immunosuppression of the TME, which may affect the chemotherapy outcome.

### 3.3 Validation of XBP1^+^ B and ACTB^+^ Treg signatures in an independent dataset

As mentioned above, we initially confirmed that XBP1^+^ B and ACTB^+^ Tregs were associated with chemoresistance. To further validate this finding, we investigated whether these two cell subtypes were more susceptible to chemoresistance.

**Figure 4:**
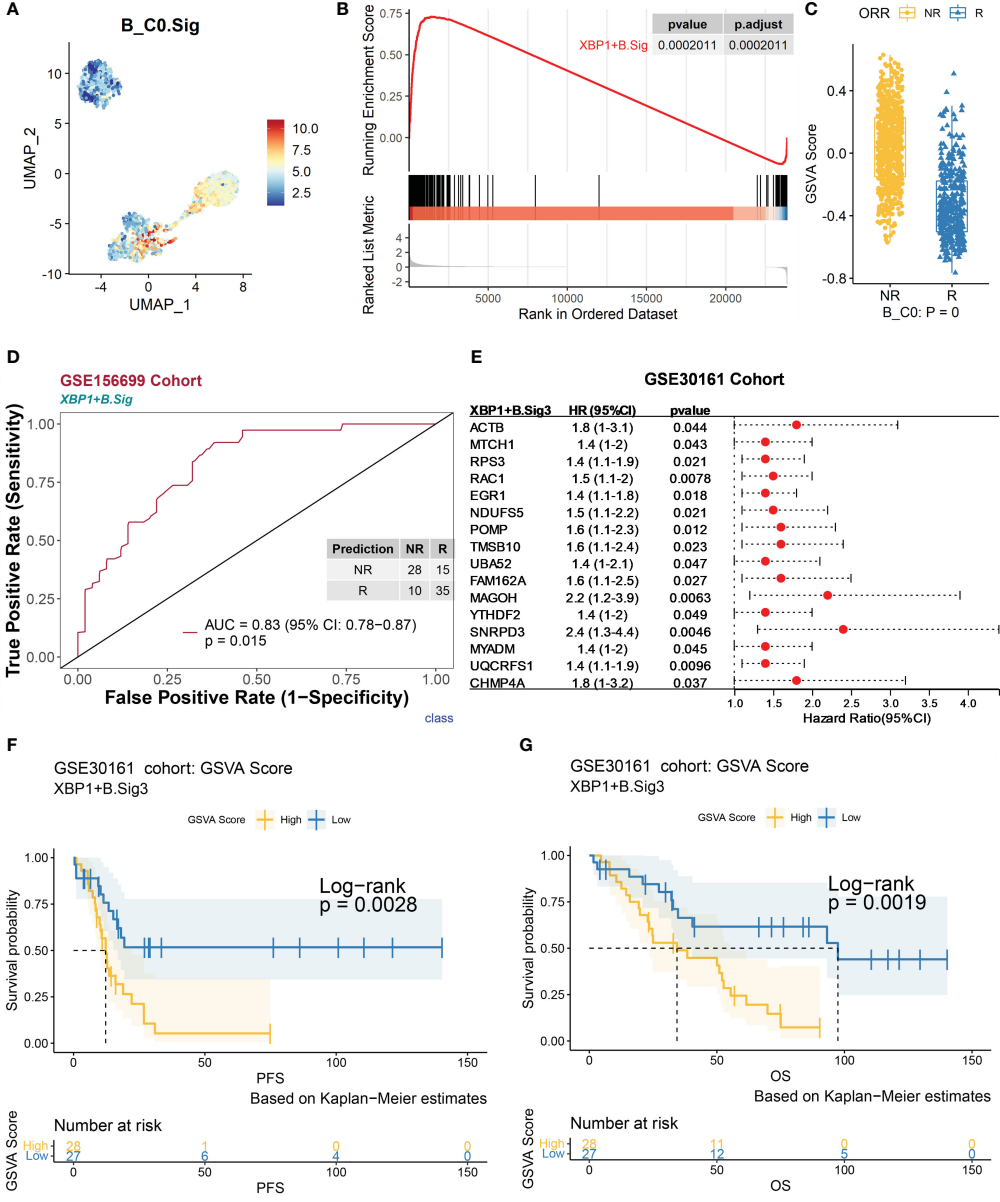
Validation of marker genes for XBP1^+^ B using an independent bulk RNA sequencing dataset. The scRNA-seq datasets GSE154600, GSE156699, and GSE30161 of B cells were analyzed. **(A)** Characterization plots of GSVA scores show that XBP1^+^ B.Sig can specifically characterize the B-cell C0 subcluster. **(B)** GSEA shows that XBP1^+^ B.Sig is significantly enriched in NR cells of the B-cell C0 subcluster. FDR adjustment of *p*-values was performed using the FDR method. **(C)** By GSVA analysis, the boxplot shows that the NR GSVA score of XBP1^+^ B.Sig is significantly higher than R in the B-cell C0 cluster. Box limits, upper and lower quartiles. Center line, median. Whiskers, 1.5 interquartile range. Points beyond whiskers, outliers. A two-sided Wilcoxon test was used to determine significance. **(D)** Prediction ability performance of XBP1^+^ B.Sig with 260 chemotherapy response markers in the GSE156699 cohort. **(E)** Univariate Cox regression analysis of genes with significant enrichment of XBP1^+^ B.Sig in NR cells of the B-cell C0 subcluster (XBP1^+^ B.Sig2) obtained from the GSEA results of XBP1^+^ B.Sig **(B)**, resulting in genes with higher prognostic risk (HR > 1) and higher significance (*p* < 0.05) of prognosis-related genes (XBP1^+^ B.Sig3) and visualized as in **(F)**. **(G)** Survival analysis of GSVA scores of XBP1^+^ B.Sig in the GSE30161 cohort (55 patients, R = 54, NR = 1). Groups were dichotomized according to median GSVA, and significance was determined using the log-rank test. Dashed line: median survival time. Color range: 95% confidence interval (CI).

The 260 marker genes of XBP1^+^ B (denoted as XBP1^+^ B.Sig) encompass the synthetic processing of cellular proteins and the regulatory process of apoptosis (**Figures 4** and **S4A**). The GSVA scores of XBP1^+^ B.Sig were significantly higher than those of other B-cell subpopulations (**Figures 4A** and **S4B**). The GSEA showed that these genes were enriched in nonresponders (**Figure 4B**), and the GSVA scores were also higher in XBP1^+^ B cells from nonresponders (**Figure 4C**). The AUC obtained for predicting chemotherapy response outcome in the GSE156699 cohort (22) (*n* = 88, R = 50, NR = 38) using XBP1^+^ B.Sig was 0.83 (*p* = 0.015, **Figure 4D**). Based on the GSEA results in **Figure 4B**, XBP1^+^ B.Sig was screened for enrichment in the gene panel. XBP1^+^ B.Sig2 (*n* = 149) was associated with differentially expressed genes in nonresponders.

Next, we analyzed the impact of XBP1^+^ B cells on the prognosis of chemotherapy patients. As determined by univariate Cox regression analysis for each gene in the XBP1^+^ B.Sig2 gene panel (**Figure 4E**), the genes that were significantly associated with patient’s prognosis were grouped as in the XBP1^+^ B.Sig3 (*n* = 16) gene panel. In the GSE30161 cohort (28) (*n* = 55, R = 54, NR = 1), the group with a low GSVA score for XBP1^+^ B.Sig3 was associated with better progression-free survival (PFS, *p* = 0.0028) (**Figure 4F**) and overall survival (OS, *p* = 0.0019) (**Figure 4G**).

Similarly, ACTB^+^ Treg.Sig contained 195 genes involved in the regulation of cell cycle and apoptosis (**Figure S4C**). Violin and feature maps of GSVA scores showed that this gene set was specific for ACTB^+^ Treg features (**Figures S5B and S4D**). In the single-cell dataset, the GSVA scores of ACTB^+^ Treg.Sig were significantly higher in nonresponders than in responders (**Figure S5D**) (**Figure S5E**). ACTB^+^ Treg.Sig predicted the response to chemotherapy in the GSE156699 cohort with an AUC of 0.82 (*p* = 0.0056) (**Figure S5F**). Based on **Figure S5D** GSEA results, ACTB^+^ Treg.Sig was screened for ACTB^+^ Treg.Sig2 (*n* = 73), a gene panel enriched in differentially expressed genes associated with nonresponders.

Next, we analyzed the effect of ACTB^+^ Tregs on the prognosis of chemotherapy patients, and achieved ACTB^+^ Treg.Sig3 using a similar strategy as B cells (**Figure S5G**). Survival analysis of the GSE30161 cohort showed that high ACTB^+^ Treg.Sig3 GSVA scores were associated with poorer OS and PFS (**Figures S5H, I**). The above results confirm that ACTB^+^ Treg shortens OS and PFS and promotes chemotherapy resistance.

### 3.4 FCN1^+^ macrophages and CCL3^+^ macrophages are associated with the chemotherapy response

As previously mentioned, some gene sets enriched in responder macrophages (Macrophages.R) had good predictive power (**Figures 2F** and **S2**; see Extended Data 2), suggesting that certain subtypes of macrophages may be associated with the chemotherapy response.

**Figure 5:**
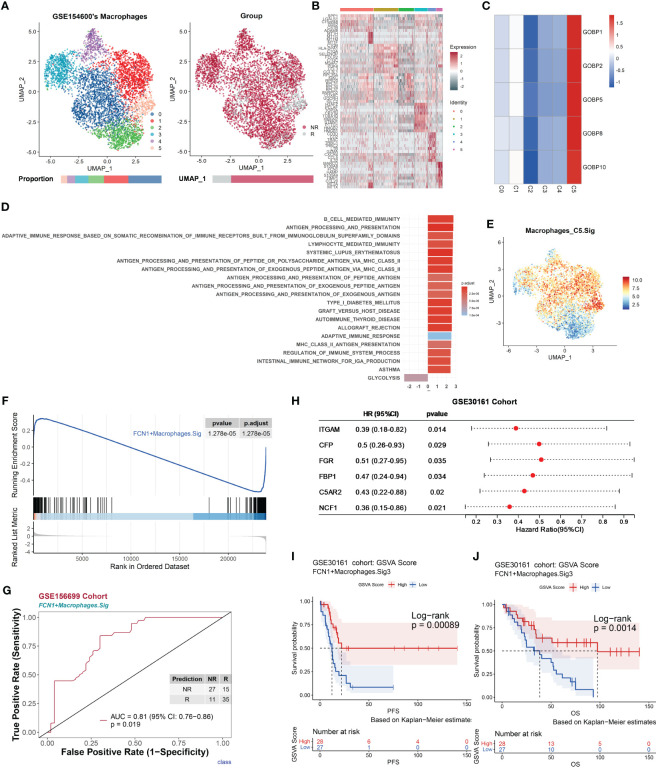
Macrophages promote antitumor immune responses, correlate with chemotherapy response, and are validated by an independent bulk RNA dataset. The scRNA-seq dataset GSE154600 of macrophages and the GSE156699 and GSE30161 cohorts were analyzed. **(A)** UMAP plot of macrophages from GSE154600. Macrophages were further divided into six clusters containing two different chemotherapy response outcomes, R and NR. Bar graphs show the proportion of cells sorted by clusters (left) and chemotherapy response (right). **(B)** Heat map of standardized expression of the top 10 specific marker genes of the macrophage-cell subpopulation of GSE154600 as determined by bilateral Wilcoxon rank sum test and FDR correction (*p* < 0.05). **(C)** Expression of GOBP gene sets with significant (*p* < 0.05) predictive power was localized by gene set variance analysis (GSVA) to identify macrophage-cell subtypes associated with chemotherapy response prediction. **(D)** Results of GOBP, Hallmark, KEGG, and Reactome enrichment analyses of the C5 subpopulation of macrophages from GSE154600. **(E)** Characterization plots of GSVA scores show that FCN1^+^ Macrophages.Sig can specifically characterize macrophage-cell C5 subclusters. **(F)** GSEA shows that FCN1^+^ Macrophages.Sig was significantly enriched in NR cells of the macrophage-cell C5 subcluster. FDR adjustment of *p*-values was performed using the FDR method. **(G)** Prediction ability performance of FCN1^+^ Macrophages.Sig with 260 chemotherapy response markers in the GSE156699 cohort. **(H)** Univariate Cox regression analysis of genes with significant enrichment of FCN1^+^ Macrophages.Sig in R cells of the macrophage-cell C5 subcluster (FCN1^+^ Macrophages.Sig2) obtained from the GSEA results of FCN1^+^ Macrophages.Sig **(F)**, resulting in genes with higher prognostic risk (HR < 1) and higher significance (*p* < 0.05) of prognosis-related genes (FCN1^+^ Macrophages.Sig3), and visualized as in **(I)**. **(J)** Survival analysis of GSVA scores of FCN1^+^ Macrophages.Sig3 in the GSE30161 cohort (*n* = 55, R = 54, NR = 1). Groups were dichotomized according to median GSVA, and significance was determined using the log-rank test. Dashed line: median survival time. Color range: 95% confidence interval (CI).

The UMAP of macrophage cells (NR.cells = 4286, R.cells = 970) in the GSE154600 dataset revealed six clusters (**Figures 5A, B**). Typical marker genes of macrophages were highly expressed in all clusters (**Figure S7A**). In Macrophages.R, there were five gene lists with AUC *p*.adjust < 0.05 (**Figure 2A**), and they were uniquely highly expressed in subcluster 5 (Macrophages_C5, NR.cells = 119, R.cells = 212) (**Figures 5C** and **S7B**). Macrophages_C5 highly expressed proinflammatory-related genes, including MARCO (64), FCN1, S100A8, S100A9 (65, 66), and CCL20 (67) (**Figures 5B** and **S7C**). GSEA showed that Macrophages_C5 promotes immune responses through multiple pathways, including MHC II antigen processing and presentation, and immunomodulation (**Figure 5D**, see Extended Data 3). We annotated Macrophages_C5 as FCN1^+^ macrophages, due to the high expression of Macrophages_C5 for proinflammatory phagocytosis-related genes.

To validate the role of macrophages in chemotherapy, the FCN1^+^ Macrophages.Sig gene panel was constructed based on its marker genes. The gene panel consists of 255 genes, all of which represent characteristic pathways such as antigen processing and presentation and immune defense responses (**Figure S7D**), with a good characterization of FCN1^+^ Macrophages.Sig specificity (**Figures 5E** and **S7E**) and was significantly enriched in responders (**Figures 5F** and **S7F**). The FCN1^+^ Macrophages.Sig was used to predict chemotherapy response outcomes in the GSE30161 cohort with an AUC of 0.81 (*p* = 0.019) (**Figure 5G**). Based on **Figure 5F** GSEA results, FCN1^+^ Macrophages.Sig was screened for FCN1^+^ Macrophages.Sig2 (*n* = 64), a gene panel enriched in differential genes associated with nonresponders.

Next, we analyzed the effect of FCN1^+^ macrophage cells on the prognosis of chemotherapy patients. Univariate Cox regression analysis was performed for each gene in the gene panel FCN1^+^ Macrophages.Sig2; we obtained a gene panel of FCN1^+^ Macrophages.Sig3 (*n* = 6) that was significantly associated with the patient’s prognosis (**Figure 5H**). Survival analysis of the GSE30161 cohort (*n* = 55, R = 54, NR = 1) showed a high level of FCN1^+^ Macrophages.Sig3 in GSVA scores that was associated with significantly better PFS (*p* = 0.00089) (**Figure 5I**) and OS (*p* = 0.0014) (**Figure 5J**).

Similarly, the UMAP of macrophage cells (NR. cells = 412, R. cells = 1,322) in the GSE165897 dataset showed five subpopulations (**Figure S6A**). Marker genes typical of macrophages were highly expressed in all subpopulations (**Figure S8A**). In Macrophages.R, there were 10 gene sets with AUC *p*.adjust < 0.05 (**Figure S2E**), which were highly expressed in subcluster 1 (Macrophages_C1, NR.cells = 412, R.cells = 1,322) (**Figures S6C and S8B**). Macrophages_C1 highly expressed chemotaxis-related genes (68), including CCL3, CCL4, CCL20, and CCL3L3, among others (**Figures S6B and S8C**). GSEA showed that Macrophages_C1 promoted immune responses through multiple pathways, including chemotaxis and intercellular signaling (**Figure S6D**, see Extended Data 3). We annotated Macrophages_C1 as CCL3^+^ macrophages due to the high expression of Macrophages_C1 for chemotaxis-related genes.

To validate the role of macrophages in chemotherapy, a gene panel of CCL3^+^ Macrophages.Sig was constructed based on its marker genes. This gene panel consists of 322 genes, all of which represent characteristic pathways such as chemotaxis and immune response (**Figure S8D**) and were significantly enriched in responders (**Figures S6E and S8E**), and GSVA scores were also higher in responders (**Figure S6F**). CCL3^+^ Macrophages.Sig was used to predict chemotherapy response outcomes in the GSE156699 cohort with an AUC of 0.81 (*p* = 0.033) (**Figure S6G**). Based on **Figure S6E** GSEA results, the gene panel CCL3^+^ Macrophages.Sig2 (*n* = 126) was further screened for the enrichment of CCL3^+^ Macrophages.Sig in responder-associated differential genes.

Next, we analyzed the effect of CCL3^+^ macrophages on the prognosis of chemotherapy patients. A significant survival-related gene panel of CCL3^+^ Macrophages.Sig3 (*n* = 6) was first obtained by univariate Cox regression analysis of the gene panel of CCL3^+^ Macrophages.Sig2 (**Figure S6H**). Survival analysis of the GSE30161 cohort (*n* = 55, R = 54, NR = 1) showed that a high level of CCL3+ Macrophages.Sig3 in GSVA scores was associated with significantly better PFS (*p* = 0.0069) (**Figure S6I**) and OS (*p* = 0.0044) (**Figure S6J**).

### 3.5 Model building and validation for chemotherapy response prediction

Because XBP1^+^ B cells and ACTB^+^ Tregs tended to impair chemotherapy effects, while proinflammatory-related macrophage cells promoted the chemotherapy response, we compared the predictive power of four cell subtypes (XBP1^+^ B, ACTB^+^ Tregs, FCN1^+^ macrophages, and CCL3^+^ macrophages) with other B cells, Tregs, and macrophage subtypes. Specifically, we selected the top 500 genes (obtained by Seurat’s FindAllMarkers) sorted by adjusted *p*-values in ascending order to identify the top 10 enriched GOBP gene sets for each cellular subtype of B, Tregs, and macrophage cells (**Figure S9**, see Extended Data 5). We then tested the predictive power of each gene set in the GSE156699 cohort using Cancerclass. Most of the gene sets associated with XBP1^+^ B, ACTB^+^ Tregs, FCN1^+^ macrophages, and CCL3^+^ macrophages had high predictive power compared to other gene sets (**Figures S9A–D**, see Extended Data 5). This provides the basis for developing a signature panel for chemotherapy outcome prediction based on these four cell subtypes.

Based on the selected set of genes with significant predictive power (*p*.adjust < 0.05) (**Figures S9A–D**), we tested the signature panel using Cancerclass according to the workflow in **Figure S13** (see Part 7 of *Materials and methods*). Based on the cluster-specific marker for four cell subtypes, we initially obtained 201 genes and then explored the relationship between these 201 genes and chemotherapy response prediction. GSEA showed that the top 20 pathways were mainly relevant to the immune response, defense response, immune system activation, and inflammatory response (**Figure S10A**). The majority of these pathways are responsible for activating or enhancing the immune response. The top 10 pathways were used to predict the outcome of the GSE156699 cohort, and these pathways had good predictive performance with AUCs between 0.75 and 0.79 (**Figure S10B**).

**Figure 6:**
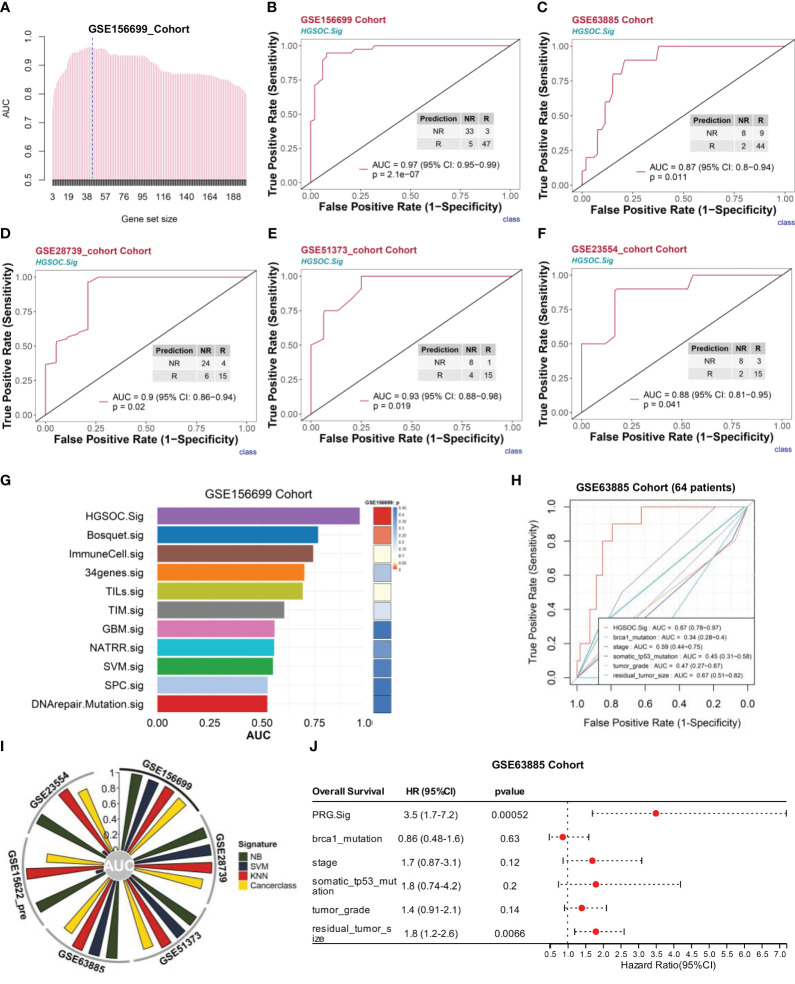
HGSOC.Sig can effectively predict the outcome of chemotherapy response in HGSOC patients. The bulk RNA-seq datasets GSE156699 (*n* = 88, R = 50, NR = 38), GSE63885 (*n* = 63, R = 53, NR = 10), GSE28739 (*n* = 50, R = 20, NR = 30), GSE51373 (*n* = 28, R = 16, NR = 12), GSE15622_Pre (*n* = 35, R = 22, NR = 13), and GSE23554 (*n* = 28, R = 18, NR = 10) were analyzed. **(A)** The bar graph shows the AUC of gene combinations and the maximum AUC per cycle (different gene number combinations). Dotted line: 43-gene combination - HGSOC.Sig. **(B)** HGSOC.Sig had a high ability to predict chemotherapy response effects in the GSE156699 cohort. **(C–F)** Good predictive ability of HGSOC.Sig in predicting chemotherapy response outcomes for the GSE63885 cohort **(C)**, GSE28739 cohort **(D)**, GSE51373 cohort I, and GSE23554 **(F)**. **(G)** Comparison of the performance (AUC vs. *p*-values) of HGSOC.Sig with the other 10 chemotherapy response signatures in the GSE156699 cohort. **(H)** Comparison of HGSOC.Sig with other clinical signatures in the GSE63885 cohort. **(I)** Verification of HGSOC.Sig using three other machine algorithms. SVM, support vector machine; NB, naïve Bayes; KNN, k-nearest neighbors. **(J)** Univariate regression analysis of HGSOC.Sig, and other clinical characteristics.

To construct a more efficient prediction model, we executed a round-robin algorithm and examined the AUCs for combinations of different numbers of genes (**Figure 6A**, see Extended Data 6). The peak of AUC appears between 38 and 57 genomic collaborations. We examined the 38-gene, 43-gene, and 57-gene combinations in the GSE156699 cohort (*n* = 88, R = 50, NR = 38). The predictive performance AUCs were 0.96, 0.97, and 0.96 (**Figures S12A, B**). In this study, we selected 43 genomic collaborations in the downstream analyses as the HGSOC chemotherapy response prediction signature (HGSOC.Sig) (**Figure 6A**, dashed line, see **Supplementary Table 2**). This response prediction signature included marker genes from XBP1^+^ B, ACTB^+^ Tregs, FCN1^+^ macrophages, and CCL3^+^ macrophages (**Figure S11A–D**). HGSOC.Sig could accurately distinguish between responders and nonresponders in the GSE156699 cohort with an AUC of 0.97 [*p* = 2.1e-07, 95% confidence interval (CI): 0.95–0.99] for the HGSOC signature (**Figure 6B**).

Subsequently, we validated the performance of HGSOC.Sig in an independent bulk RNA-seq dataset. For the GSE63885 cohort (29) (*n* = 63, R = 53, NR = 10), the AUC of this signature was 0.87 (95% CI: 0.8–0.94, *p* = 0.011) (**Figure 6C**). For the GSE28739 cohort (33) (*n* = 50, R = 20, NR = 30), the HGSOC.Sig had an AUC of 0.9 (95% CI: 0.86–0.94, *p* = 0.02) (**Figure 6D**). For GSE51373 (31) (*n* = 28, R = 16, NR = 12), the predicted performance of HGSOC.Sig had an AUC of 0.93 (95% CI: 0.88–0.98, *p* = 0.019) (**Figure 6E**). For the GSE23554 cohort (30) (*n* = 28, R = 18, NR = 10), the AUC of this signature was 0.88 (95% CI: 0.81–0.95, *p* = 0.041) (**Figure 6F**). In other cohorts, such as the NACT_Pre (34) (*n* = 6, R = 2, NR = 4), chemo_pre_RPKM cohort (35) (*n* = 20, R = 13, NR = 7), and GSE15622_pre cohort (32) (*n* = 35, R = 22, NR = 13), the AUCs for this feature were 0.79 (0.6–0.98), 0.7 (0.49–0.91), and 0.74 (0.65–0.83), respectively (**Figures S12C–E**).

To further demonstrate the predictive ability of HGSOC.Sig, we compared HGSOC.Sig with 10 other chemotherapy response characteristics previously reported in other literature (22, 23, 69–76), and the results showed that HGSOC.Sig had a higher predictive ability (**Figures 6G** and **S12F-H**). We also compared HGSOC.Sig with features that were applied in clinical applications (Brca1 mutation, TP53 somatic mutation, tumor stage, tumor grade, and residual cancer size) and showed an AUC of 0.87 for HGSOC.Sig and 0.34–0.67 for other features (**Figure 6H**).

In addition, we validated HGSOC.Sig with three more machine learning algorithms, namely, support vector machine (SVM), naïve Bayes (NB), and k-nearest neighbors (KNN). As shown in **Figure 6I**, HGSOC.Sig still performs well in most of the cohorts. These results indicate that its prediction performance is stable. The prediction of Cancerclass generated a continuous prediction score (*z*-score) according to the level of gene expression and converted it into probability (**Figure S12I**). Therefore, we converted the prediction score into the nonresponse probability to assess patients’ resistance risk after chemotherapy and estimated the risk through logical regression (**Figure S12B**). Based on tumor patients’ pretreatment RNA-seq data, we estimated the chemotherapy resistance probability and provided guidance and a reference for patients to decide whether to accept chemotherapy treatment.

Next, we investigated the link between these 201 genes and patient prognosis. Univariate Cox regression analysis of these 201 genes screened the four gene combinations (prognosis risk genes, PRG.Sig, see **Supplementary Table 2**) with the riskiest prognostic value regarding ovarian cancer patients, showing that the pretreatment GSVA score of PRG.Sig, tumor stage, tumor grade, and residual cancer size were strongly associated with poorer OS (**Figure 6J**). Multivariate Cox regression found that PRG.Sig was an independent risk factor (**Figure S12J**).

### 3.6 PPI analysis and verification of gene expression related to prediction of chemotherapy response in clinical ovarian cancer tissues

**Figure 7:**
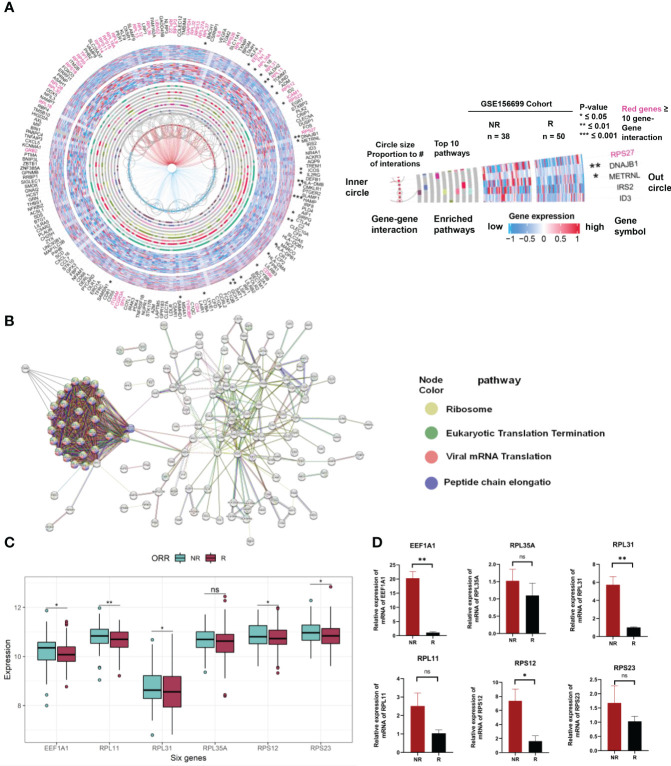
Expression and interaction analysis of 201 genes related to chemotherapeutic response prediction. **(A)** Integrating gene expression profiles, pathway enrichment analysis, and gene interactome information of differentially expressed genes between 201 chemotherapy responsive and non-responsive ovarian cancer patients associated with chemotherapy response in a circular plot. Gene symbols are listed in the outermost circles, followed by statistical significance (stars), gene expression profiles in chemo responders (external heat map of 50 samples) and chemo nonresponders (internal heat map of 38 samples), the top 10 enrichment pathways (gray circles with colored bars indicating genes found in these pathways in the dataset), hub genes (red gene names with ≥ 10 interactions with other genes in the dataset), and gene–gene interactions (circle size proportional to the number of interactions). **(B)** PPI network showing the interactions of the 201 genes (interaction score = 0.7). PPI, protein–protein interaction. **(C)** Expression of EEF1A1, RPL35A, RPL31, RPL11, RPS12, and RPS23 in Merge cohort (containing GSE15622, GSE63885, GSE51373, GSE23554, GSE28739, and GSE156699). **(D)** Expression of EEF1A1, RPL35A, RPL31, RPL11, RPS12, and RPS23 in OV tumor tissues of chemotherapy responders and nonresponders by qRT-PCR. * represents *p* < 0.05, ** represents *p* < 0.01, *** represents *p* < 0.001, and ns indicates no significance.

We used a gene pathway analysis tool (MSigDB) and STRING to compare the expression and functional significance of 201 genes between patients with chemotherapy sensitivity and resistance. The analysis revealed significant differences in the expression of six hub genes between chemotherapy responders and nonresponders in the GSE156699 cohort (*p* < 0.05). These genes, including EEF1A1, RPL35A, RPL31, RPL11, RPS12, and RPS23 (**Figure 7A**), are collectively involved in ribosomal biogenesis processes such as protein translation and peptide chain elongation (**Figure 7B**). **Figure 7C** illustrates the expression levels of the hub genes within a combined dataset of 292 individuals, including GSE15622, GSE63885, GSE51373, GSE23554, GSE28739, and GSE156699. The results demonstrate an upregulated trend of these genes in NR samples compared to R samples. Specifically, EEF1A1, RPL31, RPL11, RPS12, and RPS23 exhibited statistically significant upregulation. To validate these findings, we measured the expression levels of these six genes in OV tumor tissues of chemotherapy responders and nonresponders using qRT-PCR. As shown in **Figure 7D**, EEF1A1, RPL31, and RPS12 were significantly upregulated in chemotherapy nonresponders. Although RPL35A, RPL11, and RPS23 had *p*-values greater than 0.05 after the *t*-test comparing their expression in chemo responders and nonresponders, their expression trends were significantly higher in the chemo nonresponder group than in the chemo responder group. These results were consistent with the trends observed in the analysis of public data.

## 4 Discussion

Generally, the combination of first-stage tumor reduction surgery and platinum-based chemotherapy is the standard treatment scheme for HGSOC (4). Most may initially respond but eventually result in chemoresistance with modest overall response rates to chemotherapy, and it is crucial to identify the patients who will gain the most from these treatments. However, the prediction accuracy was not high enough in previously reported predictive models (21, 77). This highlights the significance of accurate predictive biomarkers of chemotherapy response in HGSOC. Evidence suggests that the mechanism of chemotherapy resistance in HGSOC may be associated with pre-existing gene expression in chemotherapy naïve tumor cells or their microenvironment (78). The pre-existing state of the tumor immune microenvironment can influence the response of HGSOC to chemotherapy, as well as the involvement of the inflammatory TME in regulating the chemotherapy response in this tumor, and the existence of a nonresponsive immune microenvironment before chemotherapy may lead to drug resistance due to the lack of synergistic antitumor effects mediated by immune cells [78]. The TME has multiple cellular components that can regulate tumor progression and are associated with chemoresistance (7, 79, 80).

In this study, we reanalyzed two publicly available single-cell RNA-seq datasets (25, 26) to identify valid predictive immune cell subtypes and characteristics in HGSOC. We found four key cell subtypes associated with chemotherapy response: endoplasmic reticulum stress-related XBP1^+^ B cells and invasive metastasis-related ACTB^+^ Tregs contributed to chemoresistance, and proinflammatory-related macrophages (FCN1^+^ macrophages and CCL3^+^ macrophages) associated with chemotherapy response. Using cluster-specific marker genes for these four subtypes, we developed a chemotherapy response prediction signature, HGSOC.Sig. We validated the predictive power of HGSOC.Sig in multiple datasets, and assessed its performance using various modeling approaches and known risk factors. Compared to previous prediction models, HGSOC.Sig showed good predictive performance. We further selected six genes based on differential expression analysis and PPI analysis of the cluster-specific marker genes. The expression of these six genes was validated in clinical cases, using data obtained from clinical specimens.

It is well known that B cells positively regulate immune responses and inflammation by producing antibodies, and promote T-cell activation and proliferation through antigen presentation (79). However, studies have shown that B cells can sustain immune tolerance and inhibit autoimmune and inflammatory immune responses, as well as suppress immune surveillance responses during cancer by releasing anti-inflammatory mediators (e.g., IL-10) and inhibitory molecules (e.g., PD-L1) (48, 81, 82). Recent research suggests that a subpopulation of B cells, known as regulatory B cells (Bregs), suppresses antitumor immunity (83, 84). Bregs in murine tumor models and cancer patients have been shown to attenuate antitumor immunity by secreting anti-inflammatory mediators (e.g., IL-10, TGF-β, and IL-35) by suppressing T-cell immune responses and to promote tumor progression by promoting Treg production (85). In our study, the B-cell C0 cluster (XBP1^+^ B) was associated with increased IL-10 synthesis. Meanwhile, we found that XBP1^+^ B cells were associated with the ER stress response and ERAD pathway (**Figure 3**). In the TME, immune cells control tumor development through an antitumor immune response that gradually changes as the cycle of tumor regression and regeneration progresses (86), thereby subjecting tumor and immune cells to ER stress and affecting the function of immune cells (46). A tumor’s unfavorable microenvironmental conditions, such as hypoxia, hypermetabolism, and oxidative stress, can impact how the endoplasmic reticulum (ER) folds its proteins, resulting in an “ER-stressed” cellular state and the emergence of drug resistance (87). Numerous cancers, including breast cancer, pancreatic cancer, and melanoma, have been found to be influenced by the ER stress response (49). Studies have shown that DERL3 might act as an oncogenic molecule in the immunosuppressive TME by inducing the ERAD process, and DERL3 is associated with immune cell infiltration and is especially enriched in B cells (88). Our results showed that DERL3 was more enriched mainly in the B-cell C0 cluster (**Figure S3E**). Thus, XBP1^+^ B may be closely associated with a protumor effect. However, a potential association between DERL3 and B cells has never been reported, and the specific mechanism by which DERL3 is enriched in B cells remains unknown.

In cancer, Tregs downregulate antitumor immune responses and are suppressors of antitumor immunity (89). In patients with malignancies, elevated pre-treatment peripheral Treg levels have been reported to be associated with shorter PFS, while elevated Treg in blood and tumor tissue in patients with ovarian cancer, non-small cell lung cancer, and hepatocellular carcinoma is associated with poorer prognosis and higher risk of recurrence (90–92). The prevalent mechanisms by which Tregs suppress antitumor immunity are the secretion of immunosuppressive molecules, regulation of metabolic disorders, and inhibition of dendritic cell function (93). In our study, Treg subtype C2 (ACTB^+^ Treg) had high expression of proliferation metabolism and invasion metastasis-related signature genes, which are closely associated with tumor-promoting effects. Meanwhile, ACTB^+^ Tregs were also correlated with cellular metabolic activity, oxidative stress response, and oncogenic pathways. Studies have proposed that Tregs are highly activated and proliferative in animal cancer models or cancer patients and that tumor-infiltrating Tregs require metabolic reprogramming to support their function and expansion (94). Alessia Angelin also suggested that Tregs have a selective metabolic advantage in metabolically abnormal tumor milieu (95). In comparison to normal T cells, ovarian cancer-infiltrated Tregs have increased mitochondrial activity, create more intracellular ROS, and are more vulnerable to oxidative stress in the TME (96).

Macrophages play two distinct roles in the development of cancer, i.e., antitumor effects via facilitating both phagocytosis and antibody-dependent cytotoxicity (97) and pro-tumor effects through a variety of processes, such as the stimulation of cancer proliferation and angiogenesis and the suppression of immune responses (98). M1- and M2-polarized macrophages are two different states activated consecutively in the adaptive response (99, 100), and these two functionally contrasting subtypes, the former exerting antitumor immunity and the latter exerting protumoral effects, are both highly plastic and can interconvert upon changes in the TME or upon therapeutic intervention. M1-type macrophages perform phagocytosis, antigen presentation, protection against microbial cytotoxicity, and release of cytokines and complement elements, among other activities. They are involved in tissue and systemic inflammation and immunology as well as tissue rebuilding (97, 101). The two macrophage subtypes (FCN1^+^ macrophages and CCL3^+^ macrophages) in our study from different datasets highly expressed genes related to proinflammatory-related immunity and chemotaxis, while their cluster-specific signatures were enriched for pathways related to MHC II antigen processing and presentation, immune regulation, chemotaxis, and intercellular signaling. These functional properties are consistent with the role of M1-polarized macrophages in suppressing tumor progression. According to studies, patients with high-grade serous trait ovarian cancer who have macrophages with M1 functional properties have better outcomes. Additionally, the TME’s prognostic and predictive functions may have significant clinical implications and enable the early identification of patients who are likely to respond to treatment (102).

We performed differential expression analysis and PPI network analysis on 201 genes identified from cluster-specific markers based on four cell subtypes. We found that differential genes highly expressed in chemotherapy nonresponders overlapped with hub genes from the PPI analysis, resulting in six genes associated with ribosomal biogenesis processes. Blanch et al. demonstrated that overexpression of EEF1A1 specifically inhibits p53-, p73-, and chemotherapy-induced apoptosis, leading to chemoresistance (103). RPL35A can be involved in tumor progression and plays a role as a biomarker in tumor angiogenesis (104). RPL31 and RPL11 can regulate the P53 pathway and tumor growth, and RPS12 may also be associated with tumorigenesis (105–107). Our findings suggest a strong link between ribosome biogenesis and tumor chemoresistance. Meanwhile, it has been reported that excessive ribosomal biogenesis, such as increased protein synthesis and excessive translation, often leads to abnormal cell growth and proliferation. Some studies have shown that the upregulation of proteins involved in ribosome biogenesis mediates tumor development and treatment resistance in cancer models, such as the upregulation of gene expression of RPS13 (108), RPL13 (109), RPS15 (110), and RPS11 (111).

Our study investigates the link between immune cells and tumor response to chemotherapy, and proposes an effective predictive model for HGSOC chemotherapy, which provides a pathway for the development of treatment prediction models. Our approach can be used for predictive model development for various oncologic chemotherapies. However, we only identified the relationship between the four cell types mentioned and the chemotherapy response outcome; we did not elucidate their mechanisms. Therefore, future studies are needed to explore the biological mechanisms involved in the observed relationships.

## Data availability statement

The original contributions presented in the study are included in the article/**Supplementary Material**. Further inquiries can be directed to the corresponding authors.

## Ethics statement

The studies involving human participants were reviewed and approved by Ethics Committee of the Cancer Hospital Affiliated to Shandong First Medical University. The patients/participants provided their written informed consent to participate in this study.

## Author contributions

Conceptualization: JH, YMZ, and XZ; methodology: XZ, JH, JZ, and KZ; investigation: YX, KZ and YCZ; project administration: JH and YMZ; writing—original draft: YX, KZ, LG, LC, JH, XZ, YMZ and YCZ; funding acquisition: JH. All authors have read and agreed to the published version of the manuscript.
