# Supplementary material for: A chemotherapy response prediction model derived from tumor-promoting B and Tregs and proinflammatory macrophages in HGSOC
Source: Front Oncol. 2023 Jul 14;13:1171582. doi: 10.3389/fonc.2023.1171582 (PMC10382026; doi:10.3389/fonc.2023.1171582)
Supplement: Supplementary file 1 [file DataSheet_1.pdf]

Supplementary Figure

A chemotherapy response prediction model derived from tumor-promoting B and Tregs and proinflammatory macrophages in HGSOC

First Author*, Yue Xi, Kun Zheng

*** Correspondence:** Liang chen: 155338644@qq.com; Jie Hao: jhao@fudan.edu.cn; Yiming Zhang: 202189258751@sdu.edu.cn

1 Supplementary Figures and Tables

1.1 Supplementary Figures

A

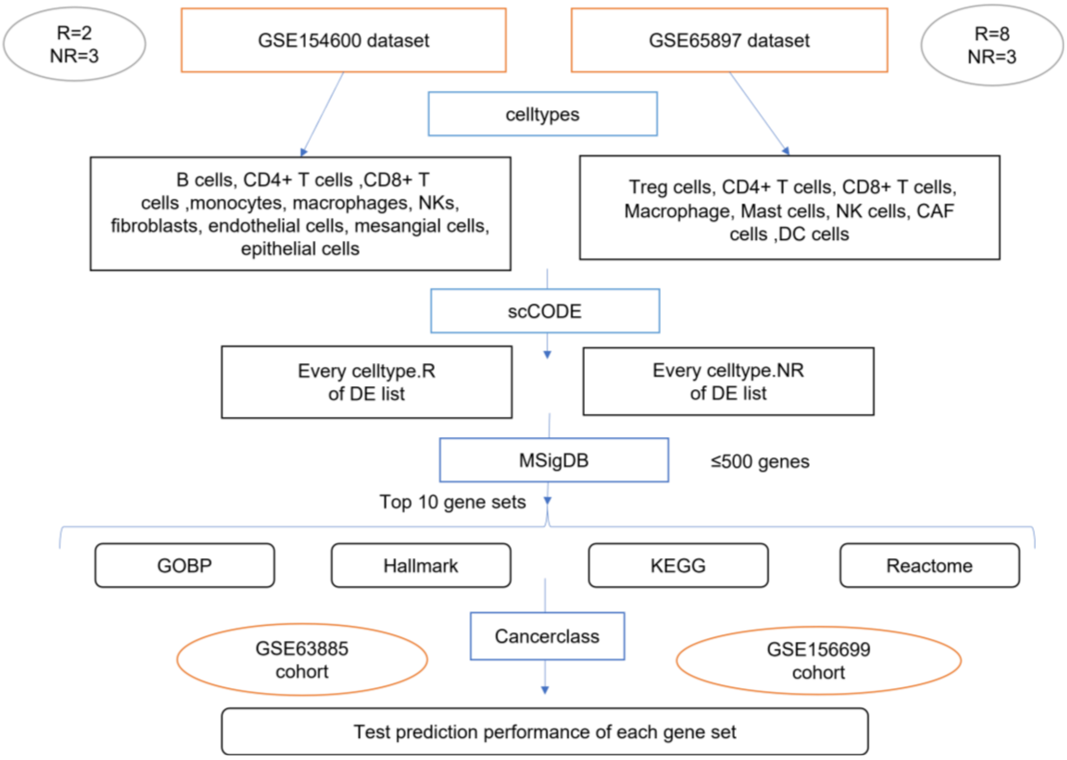

B

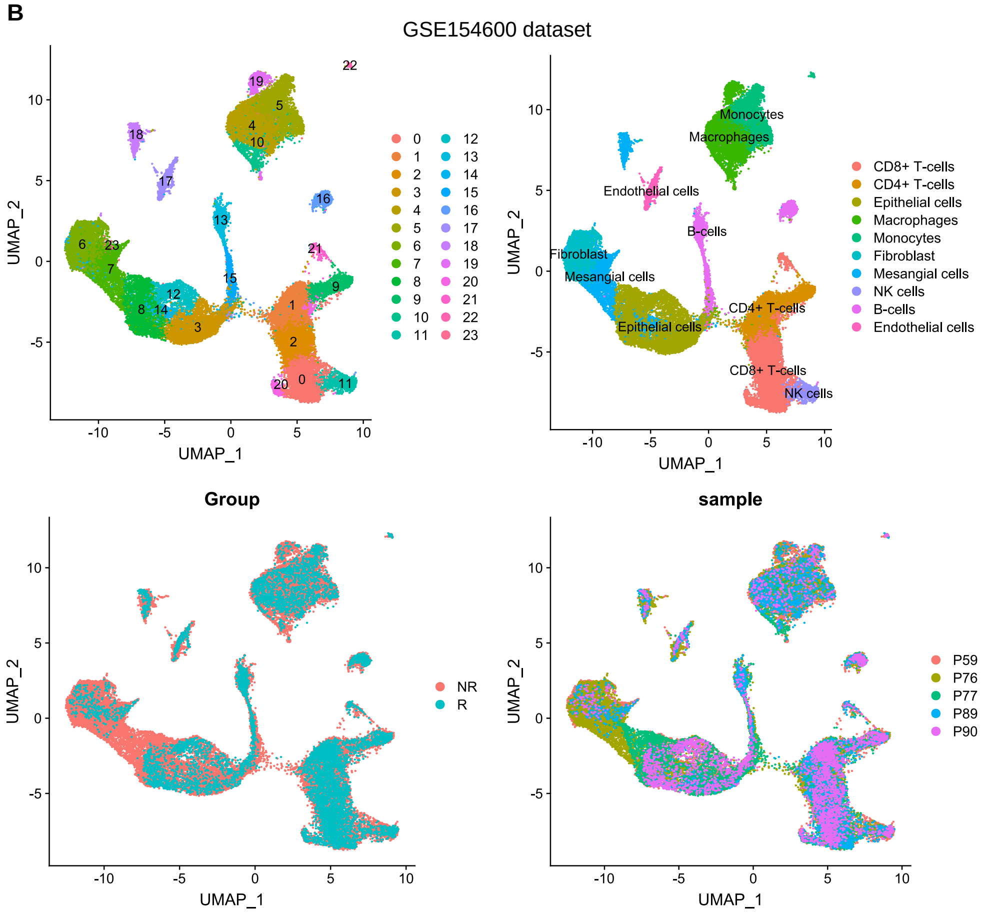
GSE154600 dataset
UMAP_1
UMAP_2
0 1 2 3 4 5 6 7 8 9 10 11 12 13 14 15 16 17 18 19 20 21 22 23
CD8+ T-cells
CD4+ T-cells
Epithelial cells
Macrophages
Monocytes
Fibroblast
Mesangial cells
NK cells
B-cells
Endothelial cells
Group
NR
R
sample
P59
P76
P77
P89
P90

C

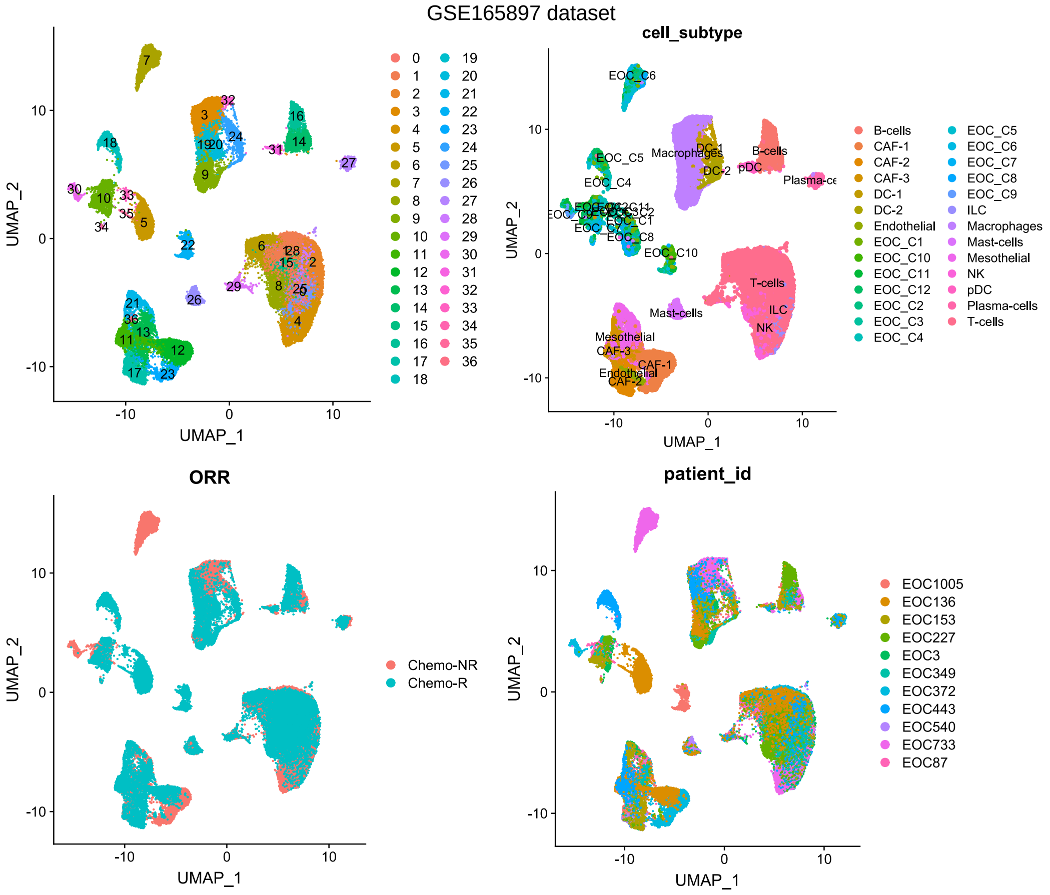
GSE165897 dataset
cell_subtype
UMAP_1
UMAP_2
0 1 2 3 4 5 6 7 8 9 10 11 12 13 14 15 16 17 18 19 20 21 22 23 24 25 26 27 28 29 30 31 32 33 34 35 36
B-cells
CAF-1
CAF-2
CAF-3
DC-1
DC-2
Endothelial
EOC_C1
EOC_C10
EOC_C11
EOC_C12
EOC_C2
EOC_C3
EOC_C4
EOC_C5
EOC_C6
EOC_C7
EOC_C8
EOC_C9
ILC
Macrophages
Mast-cells
Mesothelial
NK
pDC
Plasma-cells
T-cells
ORR
Chemo-NR
Chemo-R
patient_id
EOC1005
EOC136
EOC153
EOC227
EOC3
EOC349
EOC372
EOC443
EOC540
EOC733
EOC87

Supplementary Figure 1. Workflow on the identification of cell types and analysis of cell subtypes relevant to chemotherapy response prediction in the GSE154600 and GSE165897 datasets. (**A**) Workflow for identification of cell types associated with chemotherapy response. (**B**)Uniform manifold approximation and projection (UMAP) and aggregation were performed on the GSE154600 dataset cells. Numbers indicate the clusters obtained after unsupervised clustering (top left). Cell type categories obtained after cell annotation (top right). Distribution of chemotherapy responder (R) versus chemotherapy non-responder (NR) cells (bottom left). Distribution of cells from 5 patient samples (bottom right). (**C**) Uniform manifold approximation and projection (UMAP) and aggregation of cells on the GSE165897 dataset. Numbers indicate the clusters obtained after unsupervised clustering (top left). Cell type categories obtained after cell annotation (top right). Distribution of chemotherapy responder (Chemo-R) versus chemotherapy non-responder (Chemo-NR) cells (bottom left). distribution of cells from 11 patient samples (bottom right).

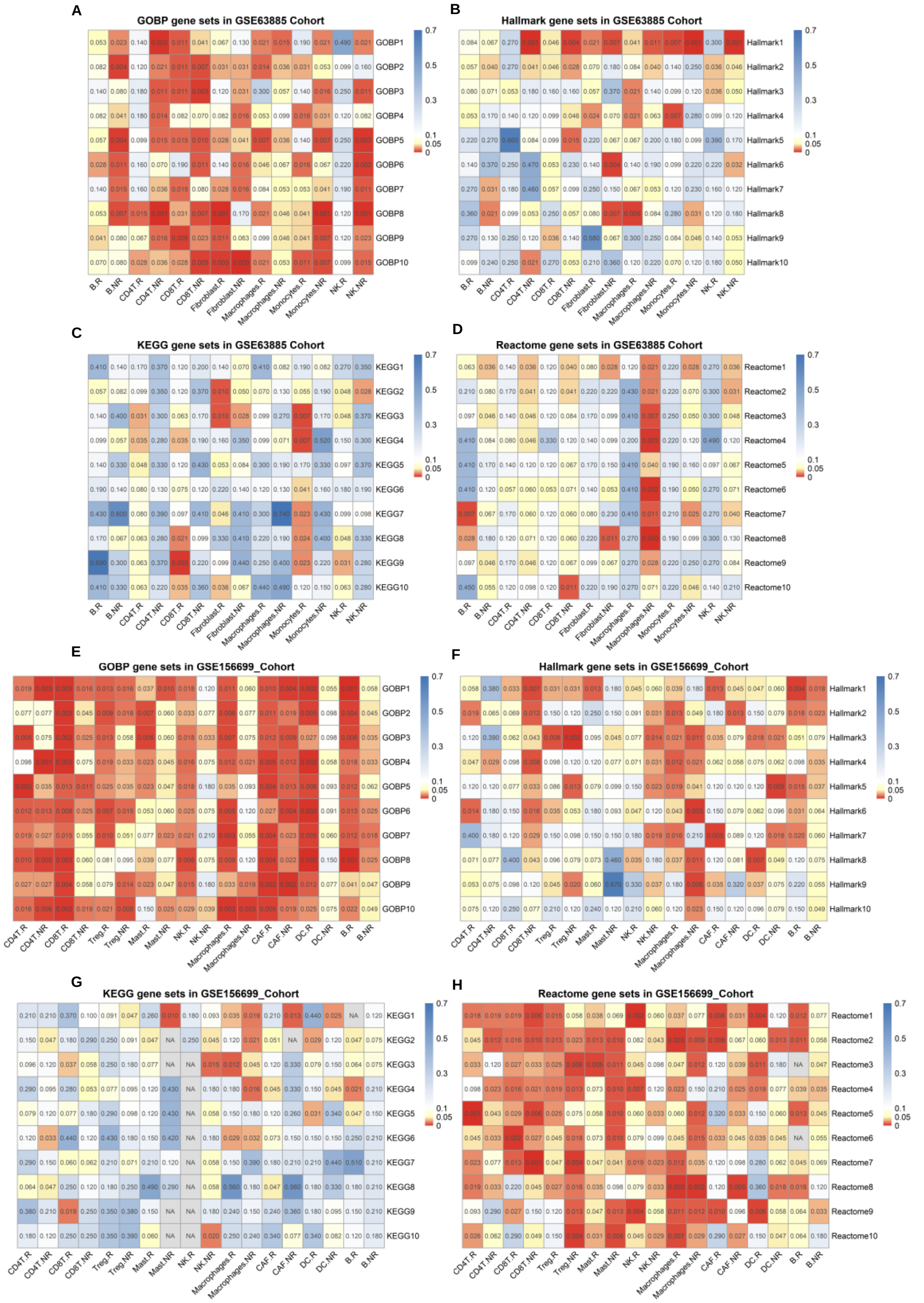

Supplementary Figure 2. The DE gene list-rich gene sets in the GSE154600 and GSE165897 datasets were used to predict chemotherapy outcomes and identify the performance of chemotherapy response-associated cell types. The GSE63885 cohort (n = 64, R = 54, NR = 10) and GSE156699 cohort (n = 88, R = 50, NR = 38) were analyzed. (A)-(D) P-values of AUC curves predicting chemotherapy response outcomes for the list of 14 DE genes in the GSE154600 dataset enriched with (A) GOBP, (B) Hallmark,

(C) KEGG, and (d) Reactome. (E)-(H) P-values of AUC curves of chemotherapy response outcomes for the list of 18 DE genes enriched with (E) GOBP, (F) Hallmark, (G) KEGG, and (H) Reactome in the GSE165897 dataset are predicted. Chemotherapy response results: R, responders; NR, non-responders.

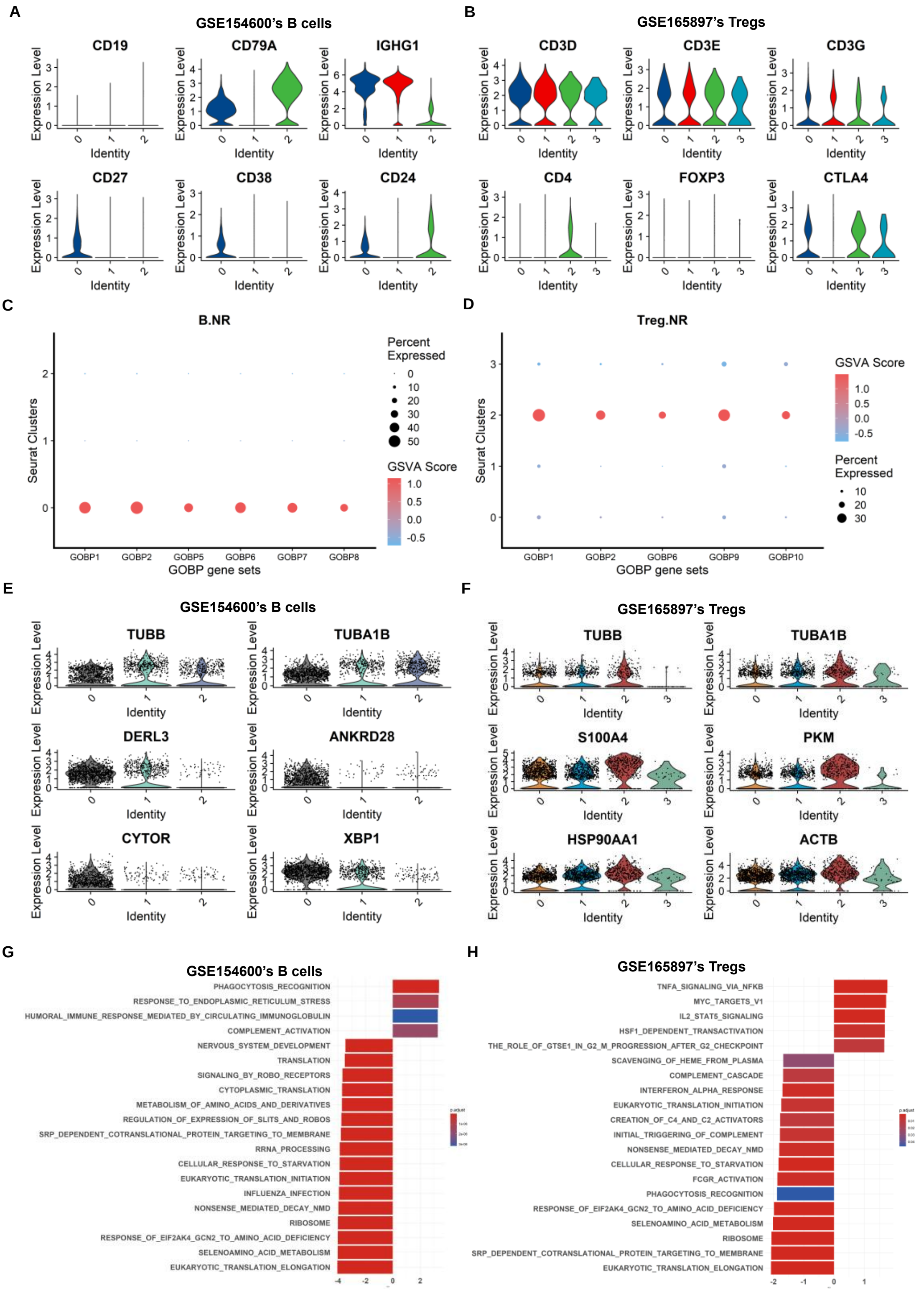

Supplementary Figure 3. B cell C0 subtype and Treg cell C2 subtype were enriched

in nonresponders and associated with chemoresistance. B cells from GSE154600 and Treg cells from the GSE165897 dataset were analyzed. Correlation with Figure 3. (A) (B) Violin plot of classical marker gene expression levels in B cells of GSE154600 and Treg cells of GSE165897 dataset. (C) and (D) Predicted gene set expression by gene set variance analysis (GSVA) locus valid (AUC p.adjust < 0.05) to identify chemotherapy response predicted associated B and Treg subtypes. Correlation with Figure 2 and Extended data 2. (E) and (F) Violin plots of proliferation-associated marker gene expression levels in B versus Treg cells. (G) and (H) Results of GOBP, Hallmark, KEGG, and Reactome analysis of B cell C0 cluster in GSE154600 and Treg cell C2 cluster in GSE165897. Normalized Enrichment Scores (NES) were listed for the top 20 pathways sorted by absolute value.

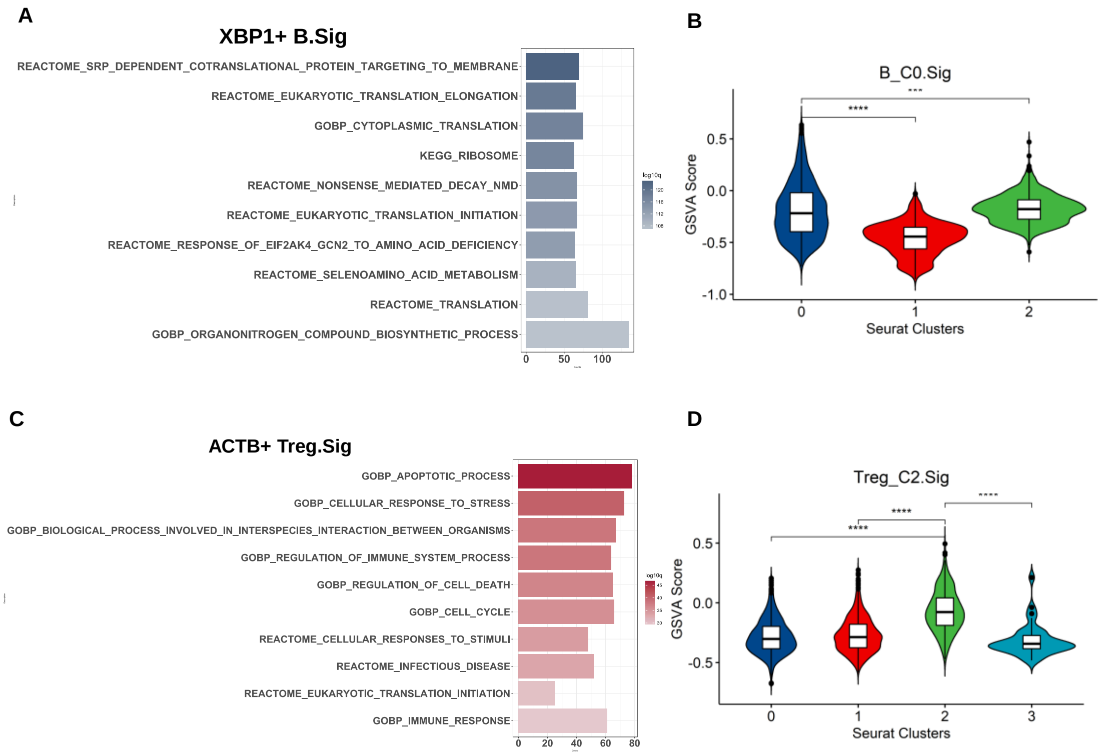

Supplementary Figure 4. (A) Results of GOBP, Hallmark, KEGG, and Reactome analyses of 260 genes in XBP1^{+} B.Sig. The top 10 pathways were ranked by FDR-adjusted p-values. (B) Violin plot of GSVA score of B_C0.Sig (XBP1^{+} B.Sig). GSVA score indicates that B_C0.Sig can specifically characterize B_C0. (C) Results of GOBP, Hallmark, KEGG, and Reactome analysis of 195 genes of ACTB^{+} Treg.Sig. The top 20 pathways are sorted by FDR-adjusted p-values. (D) Violin plot of GSVA scores of Treg_C2.Sig (ACTB^{+} Treg.Sig). GSVA scores indicate that Treg_C2.Sig can specifically characterize Treg_C2.

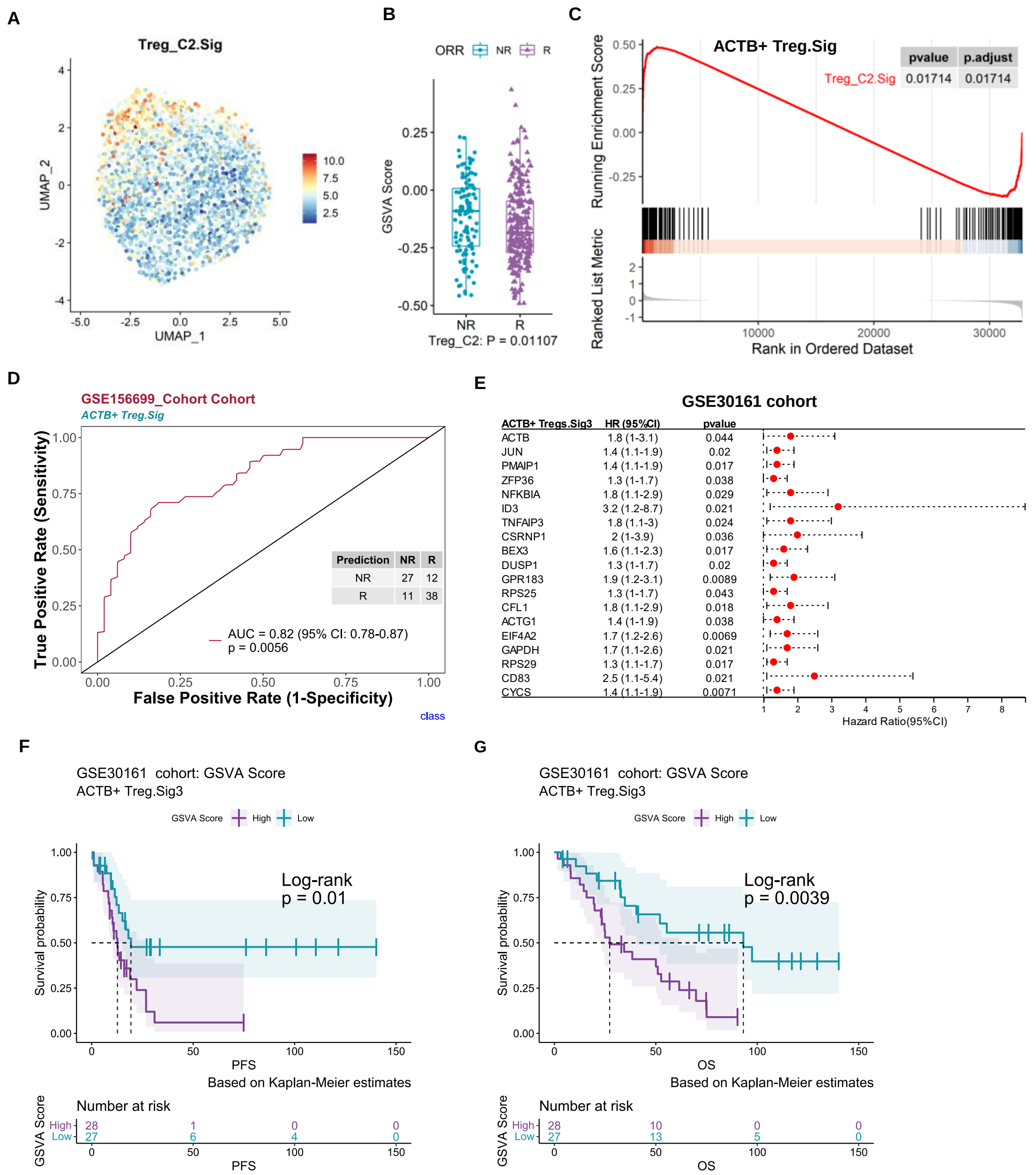

Supplementary Figure 5. Validation of marker genes for Treg_C2 using an independent Bulk RNA sequencing dataset. The scRNA-seq datasets GSE165897, GSE156699, and GSE30161 cohorts of Tregs were analyzed. (A) Characterization plots of GSVA scores show that Treg_C2.Sig can specifically characterize Treg-cell C2 subcluster. (B) GSEA shows that Treg_C2.Sig is significantly enriched in NR cells of the Treg-cell C2 subcluster. FDR adjustment of p-values was performed using the FDR method. (C) By GSVA analysis, Boxplot shows that the NR GSVA score of Treg_C2.Sig ($ACTB^+$ Treg.Sig) is significantly higher than that of R in the Treg-cell C2 cluster. box limits, upper and lower quartiles. center line, median. whiskers, 1.5 interquartile range. points beyond whiskers, outliers. a two-sided Wilcoxon test was used to determine significance. (D) Prediction ability performance of $ACTB^+$ Treg.Sig with 195 chemotherapy response markers in the GSE156699 cohort. (E) Univariate COX regression analysis of genes with significant enrichment of $ACTB^+$ Treg.Sig in NR cells

of Treg-cell C2 subcluster (ACTB$^+$ Treg.Sig2) obtained from GSEA results of ACTB$^+$ Treg.Sig (B), resulting in genes with higher prognostic risk (HR > 1) and higher significance ($p < 0.05$) of prognosis-related genes (ACTB$^+$ Treg.Sig 3), and visualized as in Figure (F) (G) Survival analysis of GSVA scores of ACTB$^+$ Treg.Sig in GSE30161 cohort (55 patients, R = 54, NR = 1). Groups were dichotomized according to median GSVA and significance was determined using the log-rank test. Dashed line: median survival time. Color range: 95% confidence interval (CI).

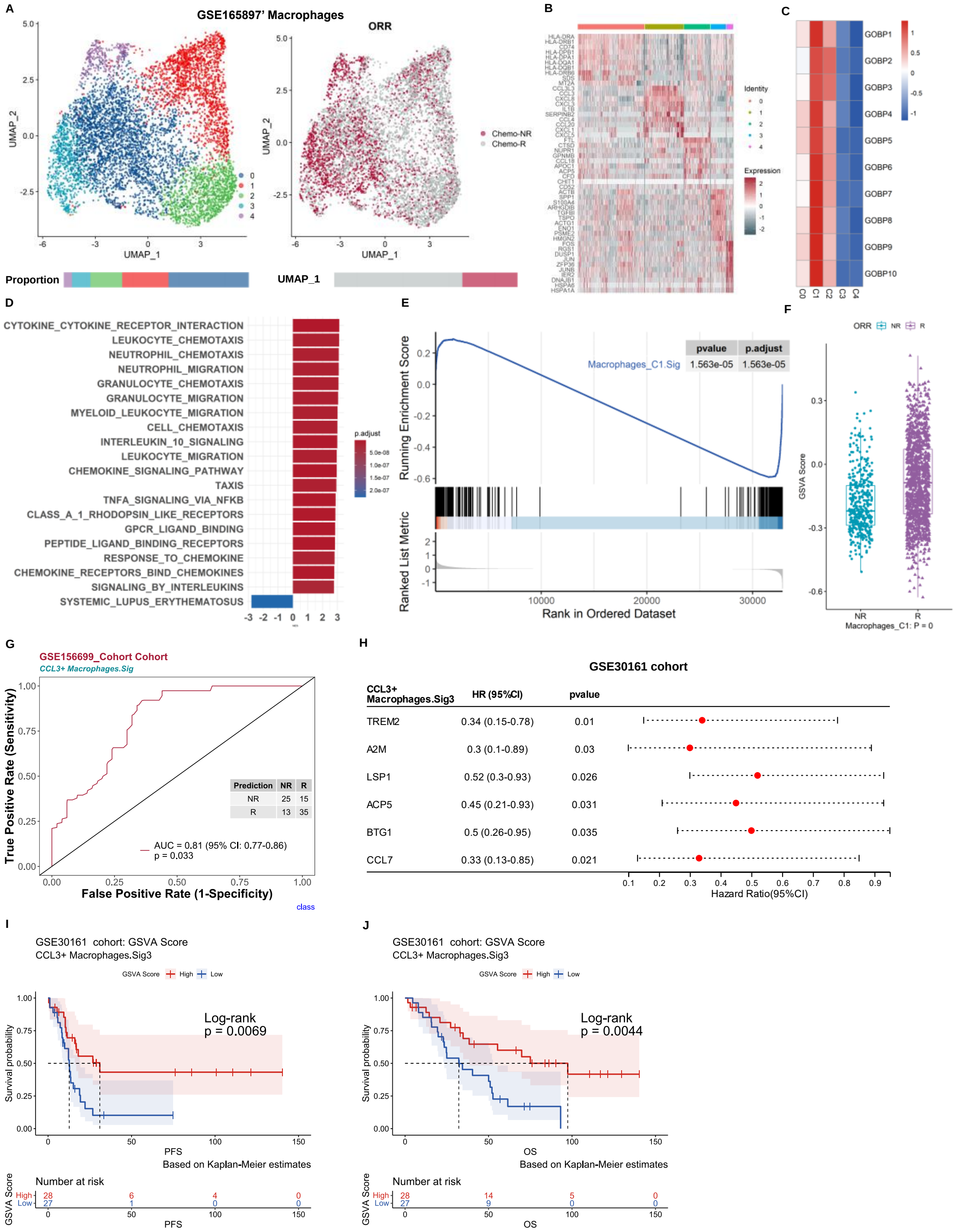

Supplementary Figure 6. Macrophages promote antitumor immune responses,

correlate with chemotherapy response, and are validated by an independent Bulk RNA dataset. The scRNA-seq dataset GSE165897 of Macrophage cells, GSE156699, and GSE30161 cohorts were analyzed. (A) UMAP plot of Macrophages from GSE165897, Macrophages were further divided into 5 clusters containing two different chemotherapy response outcomes, R and NR. Bar graphs show the proportion of cells sorted by clusters (left) and chemotherapy response (right). (B) Heat map of standardized expression of the top 10 specific marker genes of the Macrophage-cell subpopulation of GSE165897 as determined by bilateral Wilcoxon rank sum test and FDR correction ($p < 0.05$). (C) Expression of GOBP gene sets with significant ($p < 0.05$) predictive power was localized by gene set variance analysis (GSVA) to identify Macrophage-cell subtypes associated with chemotherapy response prediction. (D)Results of GOBP, Hallmark, KEGG, and Reactome enrichment analysis of the Macrophages' cluster 1 from GSE165897. (E) GSEA shows that Macrophages_C1.Sig ($CCL3^+$ Macrophages.Sig) is significantly enriched in NR cells of the Macrophage-cell cluster 1. FDR adjustment of p-values was performed using the FDR method. (F) By GSVA analysis, Boxplot shows that the R GSVA score of Macrophages_C1.Sig is significantly higher than that of NR in the Macrophage-cell cluster 1. Box limits, upper and lower quartiles. Center line, median. Whiskers, 1.5 interquartile range. Points beyond whiskers, outliers. A two-sided Wilcoxon test was used to determine significance. (G)Prediction ability performance of $CCL3^+$ Macrophages.Sig with 322 chemotherapy response markers in the GSE156699 cohort. (H) Univariate COX regression analysis of genes with significant enrichment of $CCL3^+$ Macrophages.Sig in R cells of Macrophage-cell cluster 1 ($CCL3^+$ Macrophages.Sig2) obtained from GSEA results of $CCL3^+$ Macrophages.Sig (E), resulting in genes with higher prognostic risk ($HR < 1$) and higher significance ($p < 0.05$) of prognosis-related genes ($CCL3^+$ Macrophages.Sig3), and visualized as in Figure (I) (J) Survival analysis of GSVA scores of $CCL3^+$ Macrophages.Sig3 in GSE30161 cohort (55 patients, R=54, NR=1). Groups were dichotomized according to median GSVA and significance was determined using the log-rank test. Dashed line: median survival time. Color range: 95% confidence interval (CI).

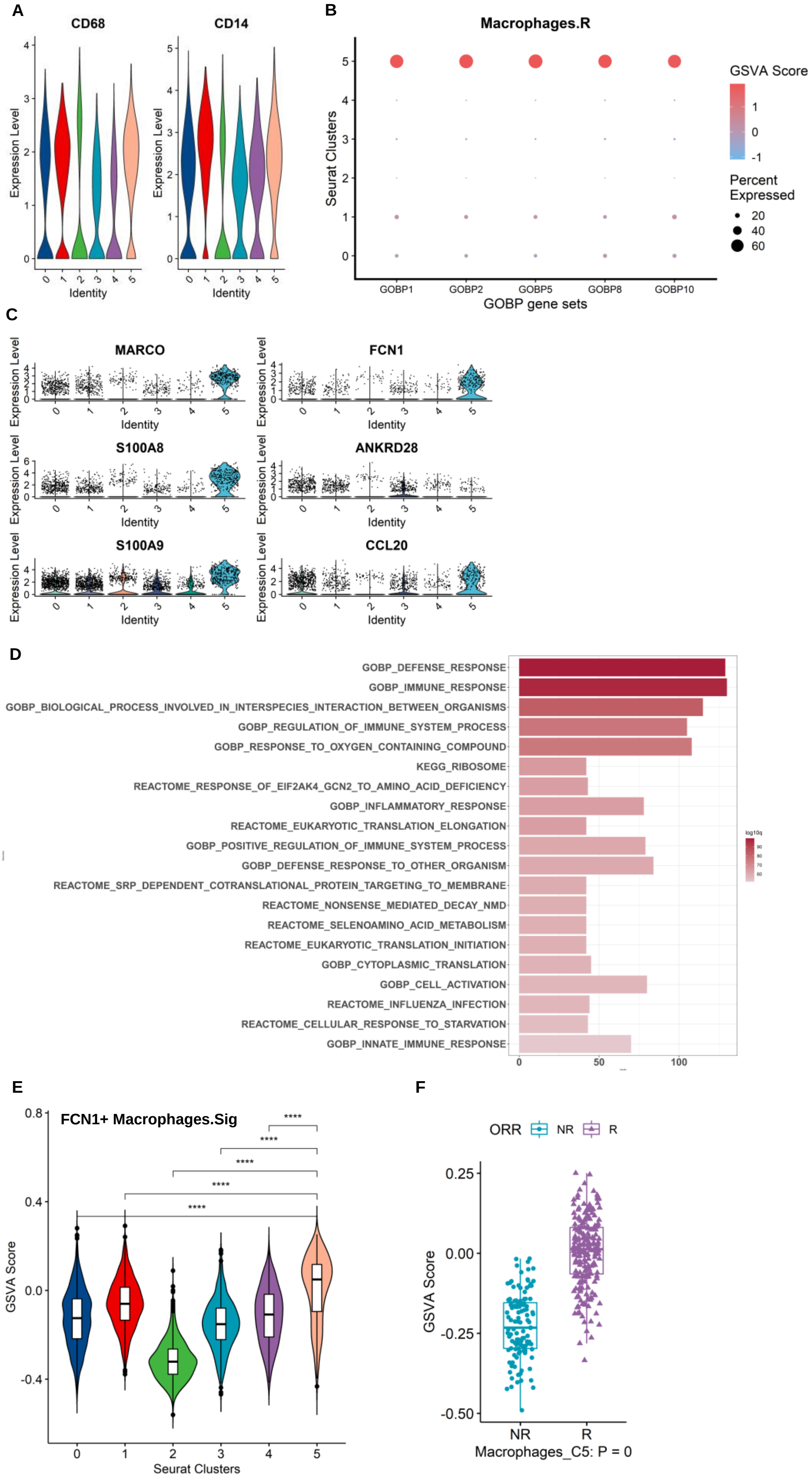
A
CD68
CD14
Expression Level
Identity
B
Macrophages.R
Seurat Clusters
GOBP1
GOBP2
GOBP5
GOBP8
GOBP10
GOBP gene sets
GSVA Score
Percent Expressed
C
MARCO
FCN1
S100A8
ANKRD28
S100A9
CCL20
Expression Level
Identity
D
GOBP_DEFENSE_RESPONSE
GOBP_IMMUNE_RESPONSE
GOBP_BIOLOGICAL_PROCESS_INVOLVED_IN_INTERSPECIES_INTERACTION_BETWEEN_ORGANISMS
GOBP_REGULATION_OF_IMMUNE_SYSTEM_PROCESS
GOBP_RESPONSE_TO_OXYGEN_CONTAINING_COMPOUND
KEGG_RIBOSOME
REACTOME_RESPONSE_OF_EIF2AK4_GCN2_TO_AMINO_ACID_DEFICIENCY
GOBP_INFLAMMATORY_RESPONSE
REACTOME_EUKARYOTIC_TRANSLATION_ELONGATION
GOBP_POSITIVE_REGULATION_OF_IMMUNE_SYSTEM_PROCESS
GOBP_DEFENSE_RESPONSE_TO_OTHER_ORGANISM
REACTOME_SRP_DEPENDENT_COTRANSLATIONAL_PROTEIN_TARGETING_TO_MEMBRANE
REACTOME_NONSENSE_MEDIATED_DECAY_NMD
REACTOME_SELENOAMINO_ACID_METABOLISM
REACTOME_EUKARYOTIC_TRANSLATION_INITIATION
GOBP_CYTOPLASMIC_TRANSLATION
GOBP_CELL_ACTIVATION
REACTOME_INFLUENZA_INFECTION
REACTOME_CELLULAR_RESPONSE_TO_STARVATION
GOBP_INNATE_IMMUNE_RESPONSE
E
FCN1+ Macrophages.Sig
GSVA Score
Seurat Clusters
F
ORR
NR
R
GSVA Score
Macrophages_C5: P = 0

Supplementary Figure 7. Macrophage's C5 subtype cells were enriched in responders and associated with chemotherapy response. Macrophage cells from the GSE154600 dataset were analyzed. Correlation with Figure 5. (A) Violin plot of classical marker gene expression levels in Macrophage cells of GSE154600. (B) Predicted gene set expression by gene set variance analysis (GSVA) locus valid (ROC p.adjusted < 0.05) to identify chemotherapy response predicted associated Macrophage subtypes. Correlation with Figure 2 and Extended data 2. (C) Violin plots of proinflammatory-related marker gene expression levels in Macrophage's C5 subtype cells. (D) Results of GOBP, Hallmark, KEGG and Reactome analyses of 255 genes in Macrophages _C5.Sig. The top 20 pathways were ranked by FDR-adjusted p-values. (E) Violin plot of GSVA score of FCN1^{+} Macrophages.Sig. GSVA score indicates that FCN1^{+} Macrophages.Sig can specifically characterize Macrophages_C5. (F) By GSVA analysis, Boxplot shows that the R GSVA score of Macrophages_C5.Sig is significantly higher than that of NR in the Macrophage-cell cluster 5. box limits, upper and lower quartiles. center line, median. whiskers, 1.5 interquartile range. points beyond whiskers, outliers. a two-sided Wilcoxon test was used to determine significance.

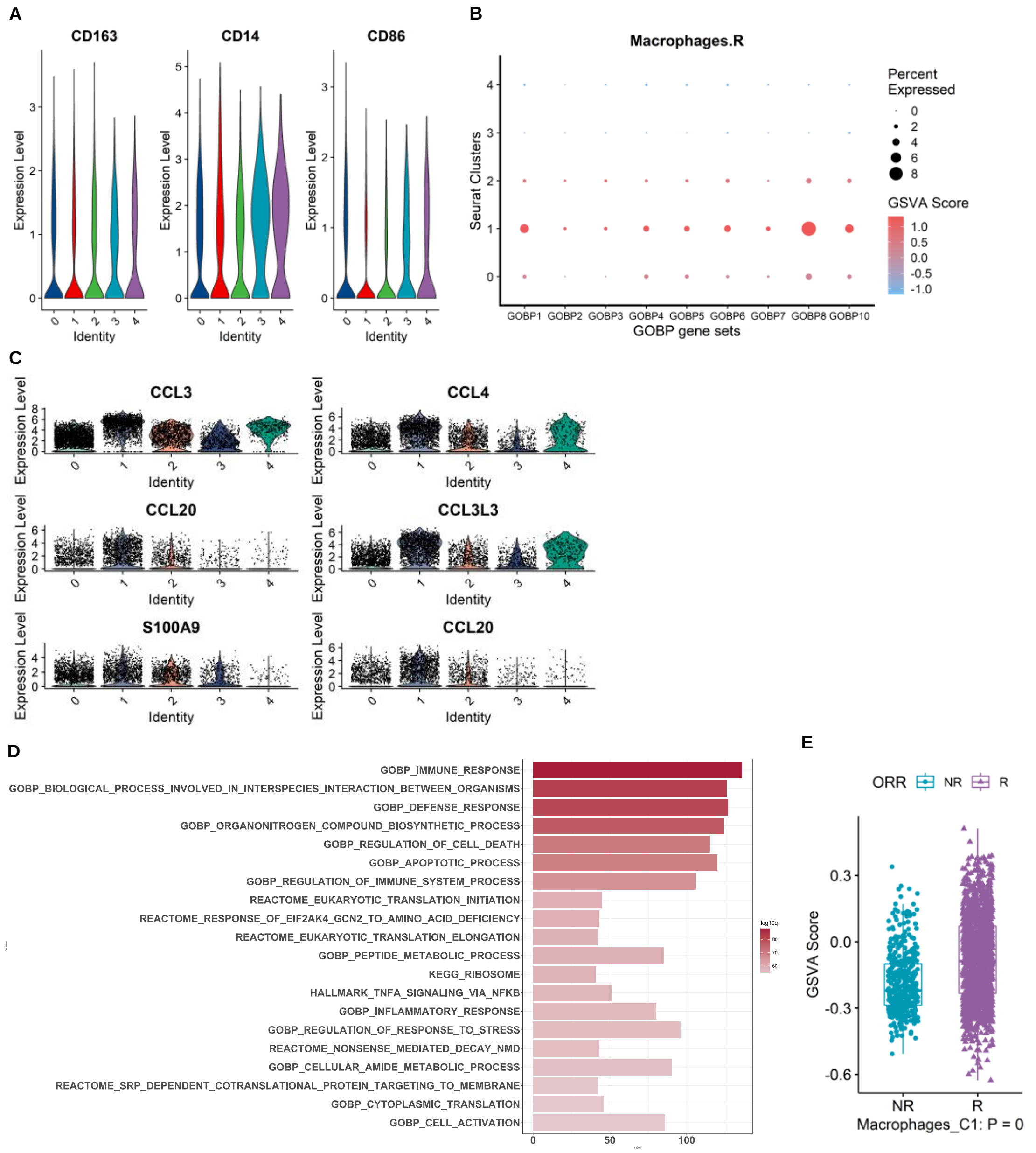

Supplementary Figure 8. Macrophage's C1 subtype cells were enriched in responders and associated with chemotherapy response. Macrophage cells from the GSE165897 dataset were analyzed. Correlation with Figure S6. (A) Violin plot of classical marker gene expression levels in Macrophage cells of GSE165897. (B) Predicted gene set expression by gene set variance analysis (GSVA) locus valid (ROC p.adjusted < 0.05) to identify chemotherapy response predicted associated Macrophage subtypes. Correlation with Figure 2 and extended data 2. (C) Violin plots of proinflammatory-related marker gene expression levels in Macrophage's C1 subtype cells. (D) Results of GOBP, Hallmark, KEGG, and Reactome analyses of 322 genes in Macrophages _C1.Sig. The top 20 pathways were ranked by FDR-adjusted p-values. (E) By GSVA analysis, Boxplot shows that the R GSVA score of Macrophages_C1.Sig is significantly higher than that of NR in the Macrophage-cell C1 cluster. box limits, upper and lower quartiles. center line, median. whiskers, 1.5 interquartile range. points beyond whiskers, outliers. a two-sided Wilcoxon test was used to determine significance.

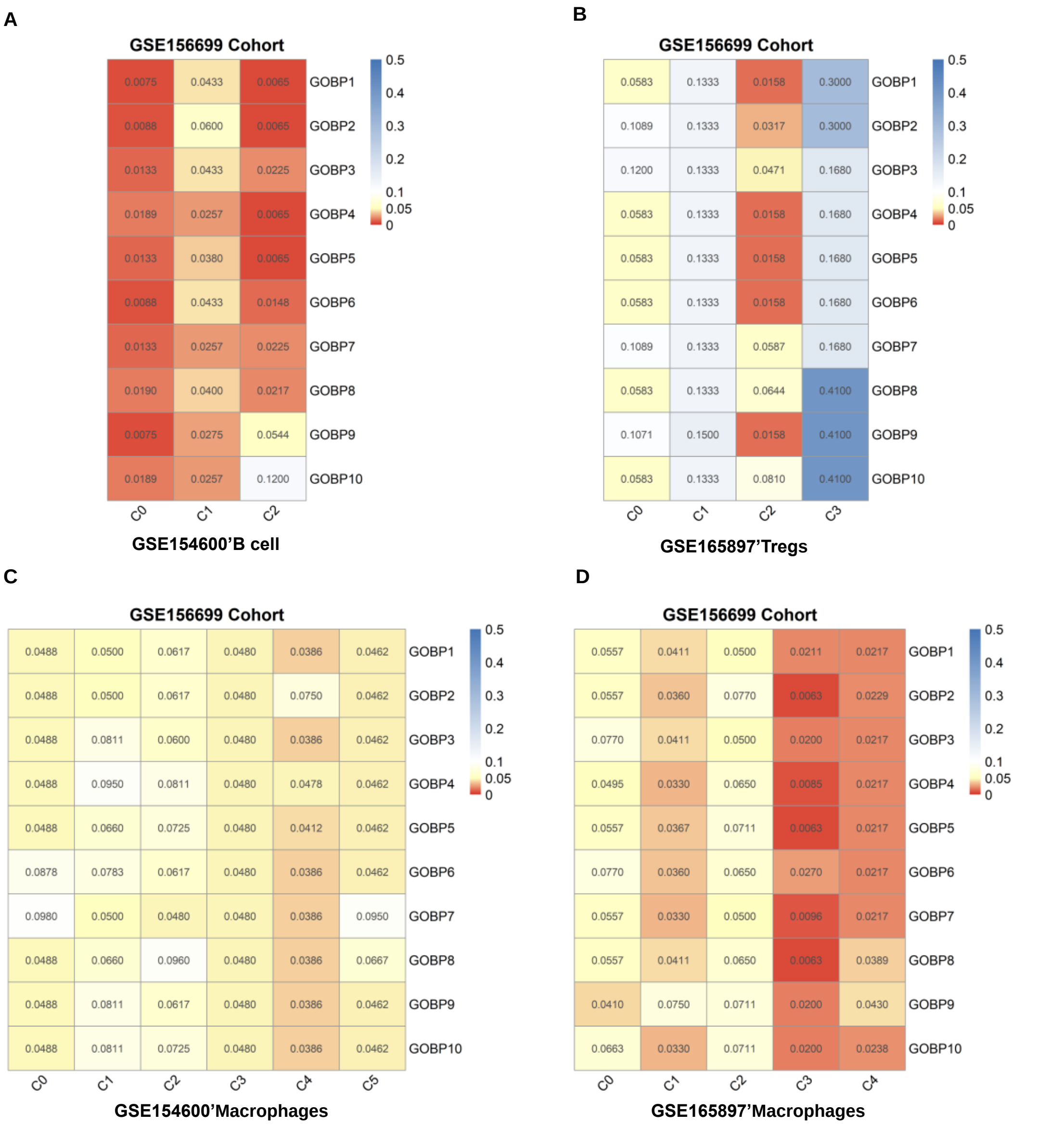

Supplementary Figure 9. Performance of gene sets enriched for four cell subtypes' cluster-specific marker genes for predicting chemotherapy response outcomes. The GSE154600 and GSE165897 datasets were analyzed, as well as the GSE156699 cohort (n.patients = 88, R = 50, NR = 38). All details are available from Extended data 4. Adjusted p-values of ROC curves to predict chemotherapy response outcomes for (A) GSE154600's B cells, (B) GSE165897 's Tregs, (C) GSE154600's macrophages, and (D) GSE165897's macrophages of cluster-specific markers enrichment in the top 10 GOBP gene lists.

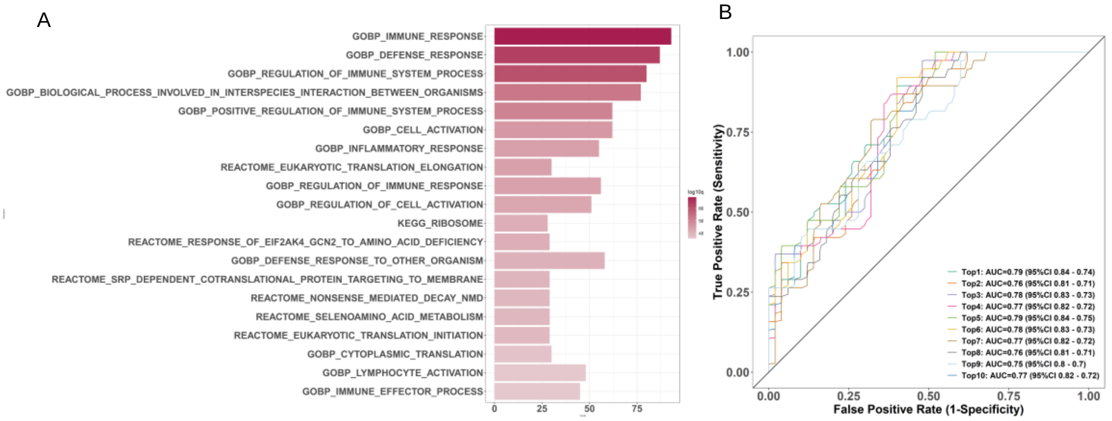

Supplementary Figure 10. 201 genes' enrichment pathways and their performance in predicting chemotherapy response outcomes. The GSE156699 cohort was analyzed. (A) Results of GOBP, Hallmark, KEGG, and Reactome enrichment analysis for 201 genes. The top 20 pathways sorted by FDR-adjusted p-value are listed. (B) Performance of the top 10 enrichment pathways in predicting chemotherapy response outcomes in the left panel (A).

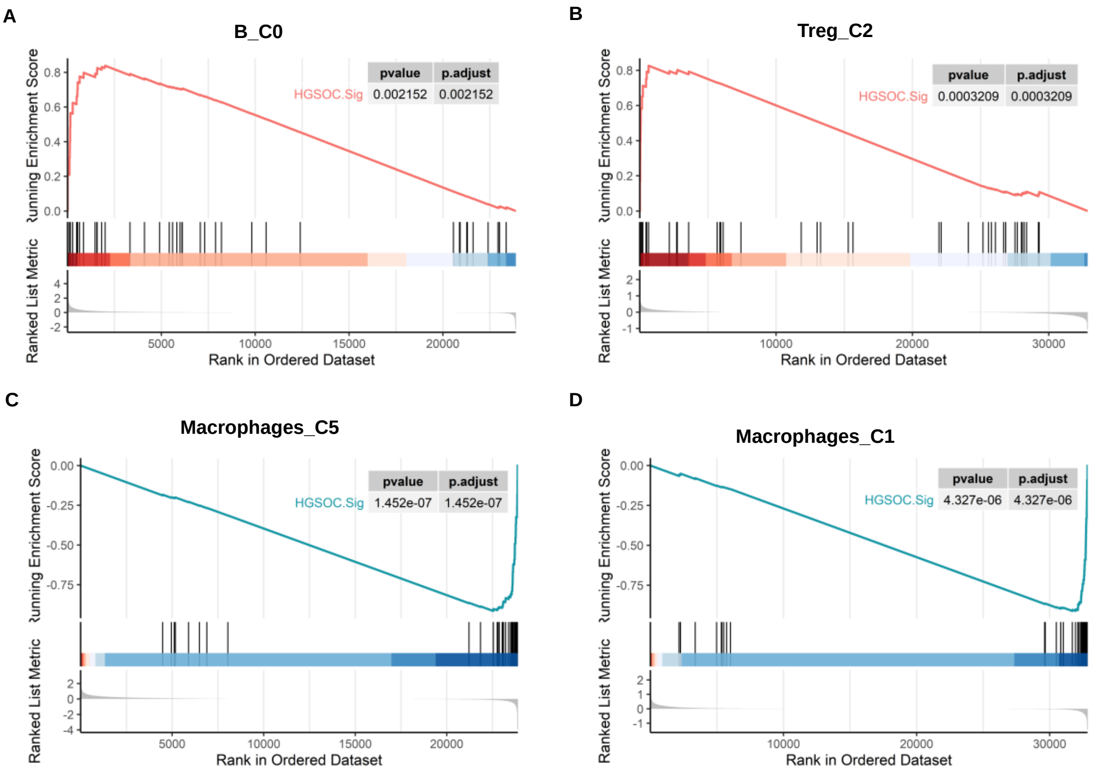

Supplementary Figure 11. Results of HGSOC.Sig marker enrichment in four cell subtypes of signature genes. HGSOC.Sig was significantly enriched in non-responders of (A) B_C0 and (B) Treg_C2 cells, and in responders of (C) Macrophages_C5 and Macrophages_C1 cells. FDR adjustment of p-values by the Benjamini-Hochberg method.

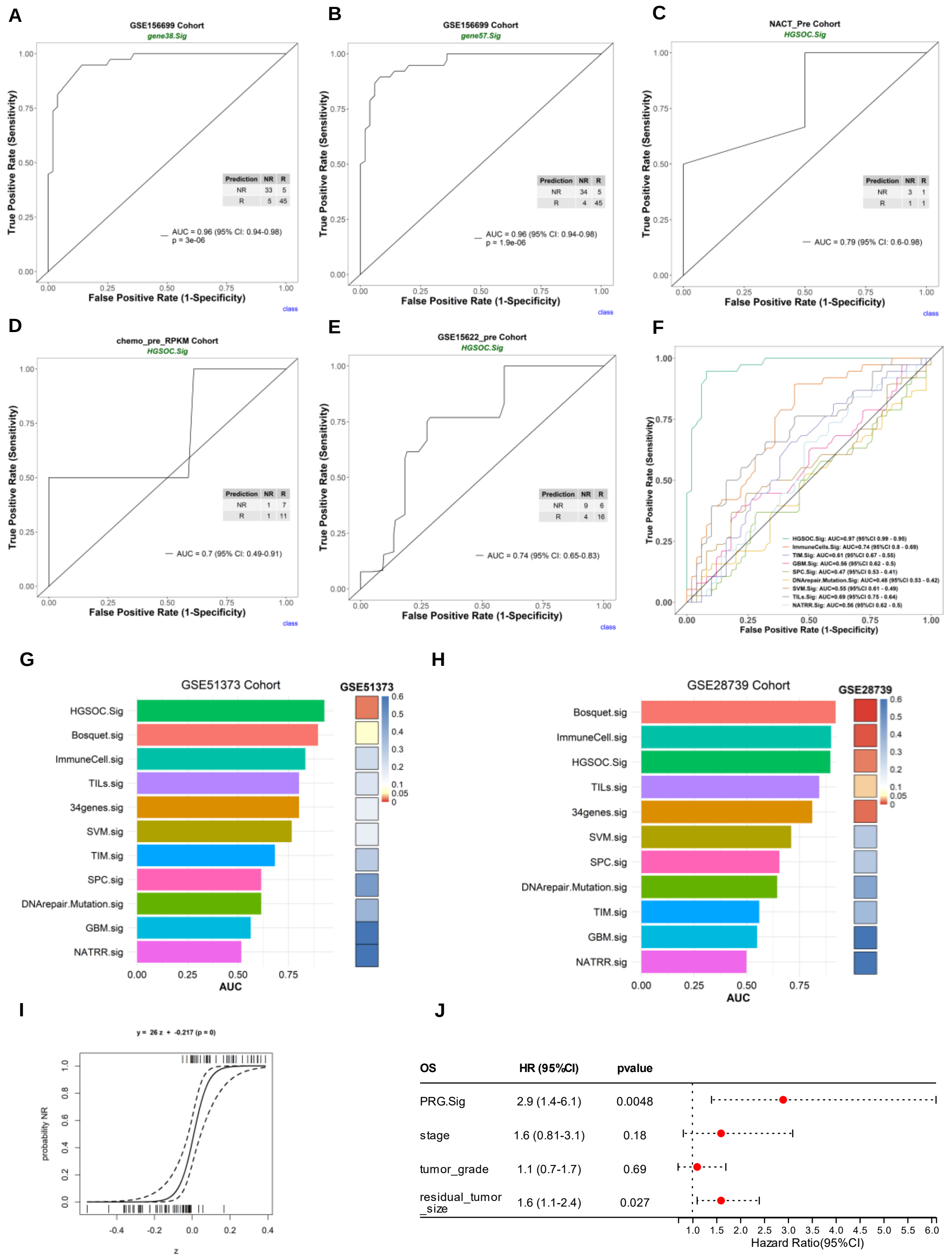

Supplementary Figure 12. Evaluate and compare the performance of HGSOC.sig and PRG.Sig. GSE156699, NACT_Pre, chemo_pre_RPKM, GSE15622_pre, GSE51373, GSE28739 and GSE63885 cohorts were analyzed. (A)(B) Predictive power of 38-gene combinations (A) and 57-gene combinations (B) in the GSE156699 cohorts. (C) (D) (E) The predictive ability of HGSOC.Sig in predicting chemotherapy response outcomes for NACT_Pre cohort (C), chemo_pre_RPKM cohort (D), GSE15622_pre cohort (E). (F-H) Results of 11 chemotherapy response prediction features in multiple ROC curves were shown. (I) Chemotherapy response prediction models can be used to estimate the

probability of chemoresistance (95% CI) by transforming the continuous prediction score (z-score) of HGSOC. Sig was performed in the GSE156699 cohort by logistic regression methods embedded in the Cancerclass R package. (J) Multiple Cox regression analysis of PRG.Sig and other clinical characteristics.

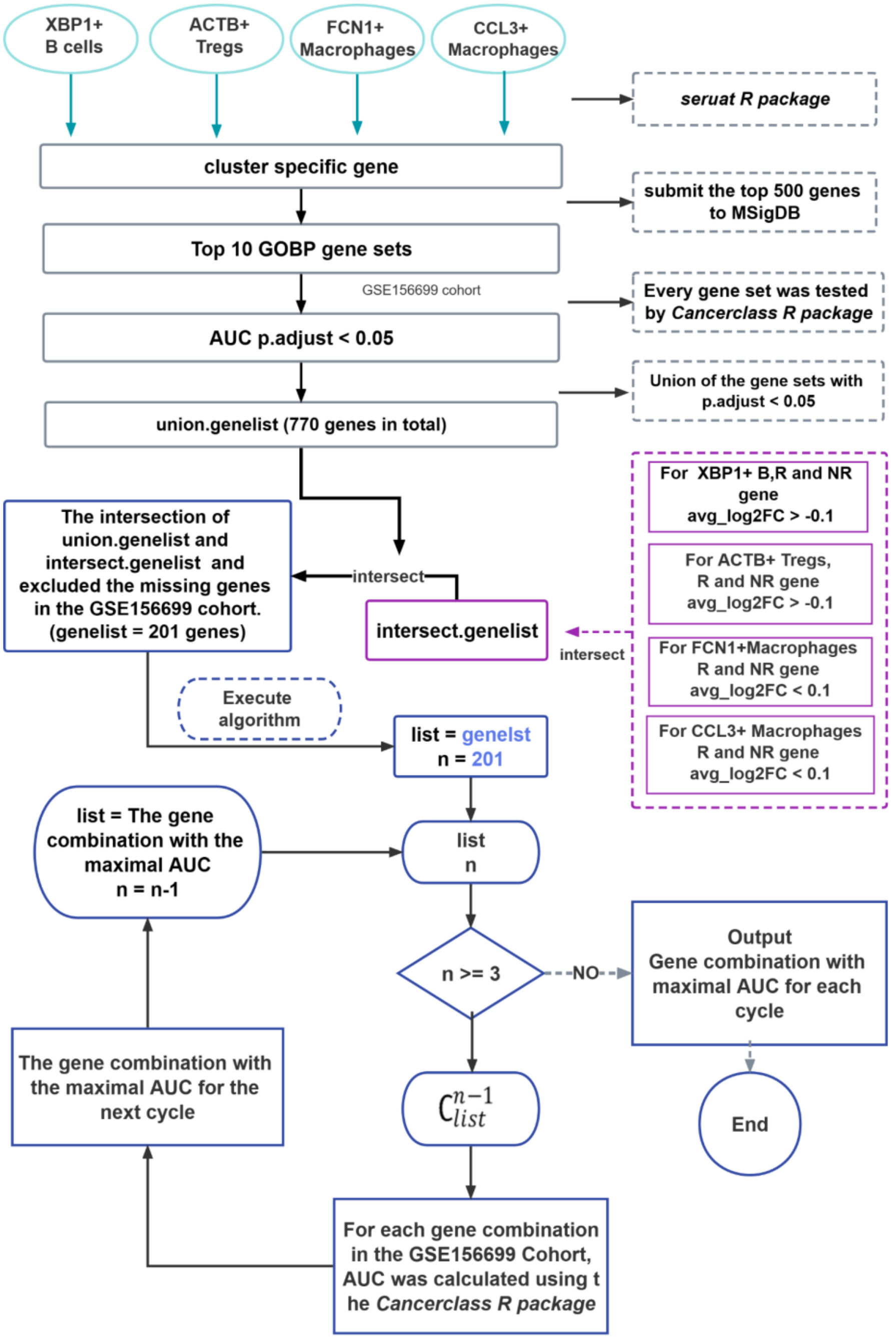

Supplementary Figure 13. Chemotherapy response prediction signature development workflow

1.2 Supplementary table

Extended data 1. scCODE results (DE gene-lists) of non-responders and responders in GSE154600 and GSE165897 dataset's cell-types.xlsx

Extended data 2. Enriched GOBP, Hallmark, KEGG, and Reactome gene sets of 38 DE gene-lists and their AUC p-values..xlsx

Extended data 3. GSEA results of GSE154600's B, GSE165897's Tregs, GSE154600's macrophages, GSE165897's macrophages..xlsx

Extended data 4. Details of XBP1+B, ACTB+ Tregs, FCN1+ Macrophages, and CCL3+ Macrophages.Sig.xlsx

Extended data 5. Top10 GOBP gene sets of GSE154600' Bs, GSE154600' Macrophages, GSE165897' Tregs and GSE165897' Macrophages enriched by cluster specific marker-genes.xlsx

Extended data 6. Gene combination with maximal AUC for each cycle (different gene-number combinations). Related to Figure 6A.xlsx

Supplementary Table 1. The scRNAseq and bulk RNAseq' metadata.xlsx

Supplementary Table 2. HGSOC chemotherapy response prediction signature (HGSOC.Sig).xlsx
